# Bone-Derived Factors: Regulating Brain and Treating Alzheimer’s Disease

**DOI:** 10.3390/biology14091112

**Published:** 2025-08-22

**Authors:** Qiao Guan, Yanting Cao, Jun Zou, Lingli Zhang

**Affiliations:** 1 School of Exercise and Health, Shanghai University of Sport, Shanghai 200438, China; 2321518016@sus.edu.cn (Q.G.); zoujun@sus.edu.cn (J.Z.); 2 College of Athletic Performance, Shanghai University of Sport, Shanghai 200438, China; 21100614@sus.edu.cn

**Keywords:** bone-brain axis, bone-derived signaling, neuro-skeletal comorbidity, bidirectional bone-brain pharmacology, whole-body vibration therapy, multi-omics biomarkers

## Abstract

In recent years, the bidirectional regulatory mechanism of the bone-brain axis has become a hotspot for interdisciplinary research. In this paper, we systematically review the anatomical and functional links between bone and the central nervous system, focusing on the regulation of brain function by bone-derived signals and their clinical translational potential. At the anatomical level, the blood–brain barrier permeability mechanism and the unique structure of the periventricular organs establish the anatomical basis for bone-brain information transmission. Innovative discoveries indicate that the bone cell network (bone marrow mesenchymal stem cells, osteoblasts, osteoclasts, and bone marrow monocytes) directly regulates neuroplasticity and the inflammatory microenvironment through the secretion of factors such as osteocalcin, lipid transporter protein 2, nuclear factor κB receptor-activating factor ligand, and fibroblast growth factor 23, as well as exosome-mediated remote signaling. Clinical studies have revealed a bidirectional vicious cycle between osteoporosis and Alzheimer’s disease: reduced bone density exacerbates Alzheimer’s disease pathology through pathways such as PDGF-BB, while AD-related neurodegeneration further accelerates bone loss. The breakthrough lies in the discovery that anti-osteoporotic drugs, such as bisphosphonates, improve cognitive function. In contrast, neuroactive drugs modulate bone metabolism, providing new strategies for the treatment of comorbid conditions. Additionally, whole-body vibration therapy shows potential for non-pharmacological interventions by modulating bone-brain interactions through the mechano-osteoclast signaling axis. In the future, it will be essential to integrate multiple groups of biomarkers to develop early diagnostic tools that promote precise prevention and treatment of bone-brain comorbidities. This article provides a new perspective on the mechanisms and therapeutic strategies of neuroskeletal comorbidities.

## Simple Summary

Bone and brain exhibit bidirectional regulatory interactions, a phenomenon termed the “bone-brain axis.” Bone-derived cells and their secreted factors actively participate in modulating neural plasticity and brain disorders. Furthermore, osteoporosis and Alzheimer’s disease (AD) form a vicious cycle: reduced bone mineral density exacerbates AD pathology, while AD-associated neurodegeneration accelerates bone loss. Therapeutically, anti-osteoporotic drugs demonstrate cognitive benefits, neuroactive agents influence bone metabolism, and whole-body vibration therapy presents non-pharmacological intervention potential. Future research should integrate multi-omics biomarkers to achieve precision prevention and treatment.

## 1. Introduction

Bone has traditionally been viewed as the primary organ responsible for providing structural support and protection, as well as participating in calcium and phosphorus metabolism and exercise. However, as research has progressed, scientific inquiry has expanded beyond single tissues or organs. The role of bone has evolved from its original functions of movement, support, and hematopoiesis to being recognized as a secretory organ in the body. It secretes bone-derived cytokines (BDCs) that affect organs beyond the skeletal system. Bone is now regarded as a potential new endocrine organ, affecting the physiological and pathological processes of the central nervous system (CNS), cardiovascular system, digestive system, and endocrine system through various bioactive cytokines secreted by its cells and bone marrow [1]. These bone cells, including bone marrow mesenchymal stem cells (BMSCs), osteoblasts (OBs), osteocytes, osteoclasts (OCs), and bone marrow-derived mononuclear cells (BMMs), play a vital role in maintaining the body’s homeostasis.

The brain, as the central control center of the human body, ensures its normal functioning. Brain damage can result in various dysfunctions and significantly impair health. Common brain disorders include Parkinson’s disease (PD), traumatic brain injuries (TBI), and subarachnoid hemorrhage (SAH), among others, which can have a substantial impact on physical health and quality of life. Therefore, comprehending the mechanisms that regulate brain function is essential for treating these disorders.

Clinical validation supports the bone and Alzheimer’s disease (AD) connection. A 2025 Japanese screening study of 8618 elderly women aged over 65 revealed that patients with low bone mineral density (BMD) had a markedly higher risk of developing AD compared to the control group, suggesting a definite link between osteopenia and AD [1]. Furthermore, studies have identified an association between low BMD and reduced gray matter volume in the hypothalamus and limbic regions [2]. These studies collectively indicate a strong correlation between low bone density and AD. Since low BMD results from disrupted bone homeostasis—a process regulated by various bone cells—it follows that different bone cell types may potentially influence AD progression. Numerous studies have demonstrated that substances secreted by bone tissue can cross the blood–brain barrier (BBB) and regulate brain function [2,3]. The interaction between bone and brain, known as the bone-brain axis, is bidirectional. The brain influences bone homeostasis through an efferent pathway, while bone regulates brain function by releasing BDCs via an afferent path [4].

This article investigates the regulation of the brain by bone, specifically examining the autotransplantation of common types of bone cells (such as BMSCs, OBs, osteocytes, and BMMs) and their secreted substances, including bone-derived factors, neurotrophins, exosomes, and extracellular vesicles. It explores brain function, regulation modes, and mechanisms. A comprehensive understanding of the functional regulation of the bone-brain axis and overall biological regulation offers a new perspective on bidirectional bone-brain regulation. It provides a framework for the targeted development of bone-derived secretory factors for treating nervous system diseases, especially AD.

## 2. Anatomy of Bone-Brain Communication

The bone-brain axis operates through dedicated anatomical pathways. Emerging evidence demonstrates that two structural components—the BBB and periventricular organs—collectively establish the core framework for neuro-osseous communication via multi-tiered channel-barrier regulatory systems. This sophisticated architecture not only exemplifies fundamental structural-functional integration in mammalian physiology but also provides a critical morphological basis for deciphering molecular pathways underlying bone-brain interactions.

### 2.1. Mechanisms of Blood–Brain Barrier Penetration

The BBB is a highly specialized physiological barrier in the CNS, the core structure of which consists of blood–brain barrier endothelial cells (BCEC). The BBB is a dynamic interface that regulates intracerebral homeostasis and protects the CNS. It responds to different physiological and pathological conditions, separating neurotransmitters and neuroactive substances in the CNS, peripheral tissues, and blood [3]. It selectively regulates the movement of substances between the bloodstream and the central nervous system, protecting the brain from harmful or unwanted chemicals [4]. Only small molecules that are fat-soluble and have a molecular weight of less than 400–500 Da can effectively cross the BBB; most large molecules cannot directly penetrate the brain endothelium to enter the brain [5]. This pathway is essential for maintaining the selective permeability of the BBB and ensuring that vital nutrients and molecules can enter the brain while keeping harmful substances out [6]. Such transcellular pathways include the lipophilic pathway, the leukocyte entry pathway, adsorption-mediated transcytosis, carrier-mediated transcytosis, and receptor-mediated transcytosis (RMT) [7]. RMT is considered to be the most prominent method of transcellular motility, characterized by its high specificity, selectivity, and affinity, and has been widely used to achieve intracerebral delivery of nanoparticles [8]. Most endogenous macromolecules require RMT to enter the brain parenchyma. For example, active transcytosis is considered to be the most likely pathway for extracellular vesicles (EV) to cross the BBB [9]. Due to the cellular origin of their outer membrane, exosomes have a unique ability to cross barrier tissues, especially the blood–brain barrier, by transcytosis compared to other biomolecules [10]. Peripheral exosomes from adults, cognitively healthy older adults, and AD patients can cross the BBB to reach the brain, and both in vivo and in vitro experiments have demonstrated that peripheral exosomes can translocate across the ductal endothelium and accumulate in the brain over time [11].

### 2.2. Mechanism of Circumventricular Organs Penetration

The periventricular organs (Circumventricular Organs (CVO)) are highly vascularised and specialized regions lacking the BBB and have secretory and sensory properties located around the third and fourth ventricles. Due to their extensive vascular system and perforated capillaries, these organs represent the only region of the brain where circulating substances that do not pass through the BBB can enter directly into CNS tissue. In addition, the projections of CVO neurons to the autonomic control center and from the autonomic control center to other deep brain regions allow blood-borne substances to act on a variety of brain regions that are mainly protected by the BBB [12]. The median hypothalamic eminence is a secretory periventricular organ. When leptin is administered peripherally in mice, this adipocyte-derived hormone shows leakage from perforated capillaries in the median hypothalamic eminence, enters the cerebrospinal fluid, and activates leptin-sensitive neurons [13]. Thus, the structural specificity of the periventricular organs, connecting the brain to external organs, enhances the inter-regulation of organs throughout the body and provides diverse pathways of action for bone regulation of the brain.

In summary, the blood–brain barrier controls the entry of molecules of skeletal origin into the brain, and the periventricular organs provide a pathway for these molecules to bypass the blood–brain barrier. Through these complex anatomical structures, the skeleton and the brain can communicate closely and work together to maintain homeostasis in the organism. The synergy of these anatomical pathways not only explains the spatiotemporal specificity of bone-brain interactions but also provides new ideas for targeted intervention in metabolic-neurological comorbidities. Subsequent chapters will delve into the specific pathways and molecular mechanisms by which the skeletal cell network regulates brain function.

## 3. Bone Cell Network Signaling Modulates Neurological Pathologies

**Figure 1:**
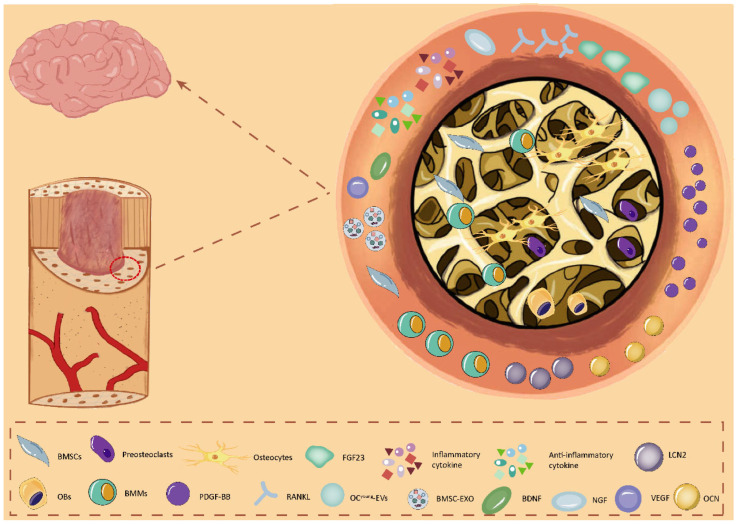
**Bone cell network signaling modulates neurological pathologies.** The principal skeletal cells implicated in brain regulation are BMSCs, OBs, osteocytes, and BMMs. BMSCs contribute to brain regulation through autologous transplantation, secretion of neurotrophic factors such as BDNF, NGF, VEGF, and various exosomes, along with the inhibition of inflammatory factors such as TNF-α, IFN-γ, IL-6, and IL-1β. OBs secrete OCN and LCN2, while RANKL, FGF23, and EVs play roles in brain regulation. BMMs modulate brain function through autologous transplantation and the secretion of PDGF-BB by preosteoclasts.

Bone’s regulation of the brain involves its participation in the physiological and pathophysiological processes of the brain through various types of skeletal cells, which secrete various bioactive cytokines. For instance, BMSCs, OBs, osteocytes, and BMMs secrete and express diverse trophic factors. Additionally, these cells secrete factors, exosomes, and extracellular vesicles to regulate brain functions (Figure 1).

### 3.1. BMSCs Regulate Brain Function

BMSCs are among the most critical cells in bone, possessing self-renewal and multilineage differentiation potential [14]. In this article, the term ‘BMSCs’ specifically denotes cells derived from bone marrow. They are considered a promising therapeutic approach for treating numerous diseases. Several studies have demonstrated that BMSCs also participate in regulating the brain through various mechanisms, including the expression and secretion of neurotrophic factors [15] and exosomes (EXOs) [16], as well as modulating the level of inflammatory factors [17]. Overall, BMSCs exhibit a wide range of functions. They can differentiate into various bone marrow cells [18] and neurons [17], support hematopoiesis, regulate immunity [18], promote tissue regeneration (including neuronal generation and synapse formation [19]), aid in wound healing, and maintain tissue homeostasis [14], and secrete neurotrophic factors such as brain-derived neurotrophic factor (BDNF), nerve growth factor (NGF) [20], and vascular endothelial growth factor (VEGF) [21], thereby promoting brain injury recovery.

#### 3.1.1. BMSCs-Derived Trophic Factors Regulate Brain Function

BMSCs can migrate to areas of nerve injury or neurodegeneration after transplantation, promoting nerve tissue repair and recovery of central and peripheral nervous system function. In an aged mouse model induced by D-galactose, intravenous injection of BMSCs resulted in the survival and migration of transplanted BMSCs to the brain. These cells differentiated into astrocytes and neurons, including choline acetyltransferase neurons, improving the early morphology and function of the aged brain [22]. This improvement may be due to the transplantation of BMSCs restoring cholinergic system function, protecting atrophic cholinergic neurons, inducing antioxidant effects, restoring neurotrophic factors, and up-regulating the levels of postsynaptic density protein 95 (PSD95) and early growth response protein 1 (Egr1) to regulate synaptic plasticity in the hippocampus, thereby enhancing hippocampal activity and cognitive function [22]. Furthermore, direct transplantation of BMSCs into the hippocampus of mice with ethanol-induced dementia significantly improved their learning and memory function. The transplantation of BMSCs inhibited neuronal apoptosis, increased superoxide dismutase (SOD) activity in the hippocampus, and significantly increased the protein level of BDNF in the hippocampus, thereby ameliorating alcohol-induced hippocampal damage [15]. Whether in vivo or in vitro, under suitable conditions, BMSCs can differentiate into neurons, astrocytes, and oligodendrocytes, thereby regulating brain function [17]. Additionally, intravenous injection of BMSCs into diabetic mice significantly improved diabetes-induced cognitive dysfunction by repairing damaged neurons and astrocytes [23]. Following infarction, the transplantation of BMSCs in mice increased neuronal and axonal density and vascular density significantly, accompanied by locally restored blood flow [24]. This effect may be related to factors secreted in ischemic tissues. For example, stromal cell-derived factor 1 (SDF-1) and its receptor, C-X-C chemokine receptor type 4 (CXCR4), induce stem cell homing [25]. SDF-1 is present around damaged brain tissue, while CXCR4 is widely expressed on BMSCs. Intracerebral injection of SDF-1 or overexpression of CXCR4 can promote the migration of transplanted BMSCs to ischemic lesions [26].

Traditionally, the treatment of nervous system diseases with BMSCs involves direct stem cell replacement of damaged tissue. However, in the treatment of stroke in mice with mesenchymal stem cell (MSC) transplantation, it was observed that only a minimal number of transplanted cells could be detected in the brain, with a minimal probability of expressing neuronal markers [27]. This suggests that BMSCs are less likely to act through tissue replacement and more likely through the secretion of trophic factors that directly or indirectly contribute to functional recovery after stroke [28]. For instance, the transplantation of BMSCs can reduce the number of apoptotic cells by activating trophic factor signaling pathways [29]. Neurotrophic factors control the CNS by regulating cell proliferation, survival, and differentiation through molecular signaling pathways [30]. Furthermore, studies have confirmed that BMSCs can produce neurotrophic factors such as BDNF and NGF through paracrine signaling after transplantation. NGF and VEGF promote angiogenesis and neurogenesis, enhancing endogenous neurogenesis, migration, and survival of neuroblasts, thereby promoting their maturation and the recovery of their functions, which are essential for neuronal survival and brain protection. This indicates a strong potential for treating ischemic stroke (IS) [15].

##### BMSCs-Derived BDNF Regulates Brain Function

BDNF is widely distributed in the brain and regulates the apoptosis, generation, and differentiation of neurons, playing an essential role in nervous system development and maintenance [31]. It can prevent neuronal cell death after various brain injuries, such as cerebral ischemia, and promote synaptic and axonal plasticity [32]. BDNF also promotes neuronal differentiation and generation. Liu et al. [33] found that BMSCs overexpressing BDNF efficiently induced the generation of neuron-like cells, suggesting a role in BDNF-induced neural differentiation. Shichinohe et al. [34] discovered that specific subsets of BMSCs promoted the survival of host neurons around infarction by secreting BDNF in the early stage (up to four weeks) after transplantation. BDNF also exhibits anti-inflammatory and neuroprotective effects against glutamate toxicity. When BMSCs were used to deliver BDNF into the brains of allergic encephalomyelitis (EAE) mice, pro-inflammatory cytokines like interferon-γ (IFN-γ) and serum tumor necrosis factor-α (TNF-α) were inhibited. In contrast, anti-inflammatory cytokines IL-11, IL-10, and IL-4 were elevated in the CNS tissues of the BDNF transplantation group [35]. Fluorescence in situ hybridisation showed that the majority of BMSCs expressed BDNF in vitro [34]. In conclusion, BMSCs can be modulated by BDNF to regulate brain neuronal apoptosis, generation, and differentiation, and exhibit anti-inflammatory properties.

##### BMSCs-Derived NGF Regulates Brain Function

NGF is a neurotrophic factor that supports the survival and growth of nerve cells [36]. Its release in the innervation area not only ensures neuron survival [37] but also regulates axon growth and synapse formation [38] and affects the synthesis of neurotransmitters and neuropeptides [39]. NGF has therapeutic effects on the injured CNS [40]. Firstly, NGF treats the CNS by modulating the function of BMSCs. NGF treatment can improve the vitality, adhesion, and migration ability of BMSCs. In a model without NGF, BMSCs are unable to reverse oxygen and glucose deprivation effectively (OGD) to brain microvascular cells induced by injury [41]. Following NGF transfection after BMSCs transplantation in cerebral ischemic mice, NGF expression in the brain increases in BMSCs, leading to increased neural cell differentiation and synaptic expression, ultimately improving neural function. These findings suggest that increasing NGF in BMSCs contributes to brain remodeling after a stroke [36]. Additionally, NGF secreted by BMSCs can reduce the inflammatory response by down-regulating the pro-inflammatory cytokine TNF-α and inhibiting the TNF-α/RelB pathway [42]. This inhibitory effect can be abolished by NGF-neutralizing antibodies or tropomyosin receptor kinase A inhibitors [43].

##### BMSCs-Derived VEGF Regulates Brain Function

VEGF is an essential regulator of physiological angiogenesis in multiple systems [21], promoting the migration and proliferation of endothelial cells [44] and positively regulating osteogenic growth factors to stimulate osteogenesis [45]. Additionally, VEGF improves brain damage by controlling the generation of cerebral microvessels, particularly in various cerebral ischemic diseases, where it can be secreted by BMSCs [21]. In mice transplanted with BMSCs following focal cerebral apoplexy, the level of VEGF increased in the infarction area, accompanied by a significant increase in neurons, axons, and vascular density, along with locally restored blood flow [24]. Similarly, the intravenous injection of BMSCs into MCAO mice via the tail vein increased the number of Ki-67-positive and VEGF-positive cells around the infarction, leading to significant neovascularization, neurofibrils, and anastomosed blood vessels in the infarction area. This suggests that the enhanced neurogenesis from BMSCs may result from VEGF-mediated and Ki-67-mediated angiogenesis [46]. Furthermore, BMSCs transplantation in global cerebral ischemia mice revealed co-expression of BDNF and VEGF in the hippocampus and temporal cortex, significantly improving brain pathology and neurological function [47]. Zong et al. [48] noted that the expression and secretion of VEGF and BDNF were elevated in the brains of MCAO mice transplanted with VEGF-based BMSCs, leading to increased microvessel density, improved behavioral function, and increased BMSC survival. Chen et al. [49] also observed a significantly greater improvement in the cerebral infarction area in mice treated with VEGF-BMSCs compared to those that received only BMSCs from 7 to 28 days after MCAO. Moreover, Guo et al. [50] found that BMSCs transplantation up-regulated the expression of VEGF and angiopoietin-1 in brain tissue following TBI, promoting microvessel formation. Hypoxia enhanced VEGF release from BMSCs, and blocking the VEGF receptor negated the protective effects provided by hypoxia-conditioned aged human BMSCs (hBMSCs) conditioned medium against neuronal oxygen-glucose deprivation [51]. However, hypoxic preconditioned BMSCs promote myelin regeneration by regulating the pro-survival mTOR/HIF-1α/VEGF signaling pathway [52]. Therefore, transplanted BMSCs can facilitate angiogenesis in the injured brain by expressing or secreting VEGF, increasing blood perfusion in the brain, and promoting functional recovery of the brain.

**Figure 2:**
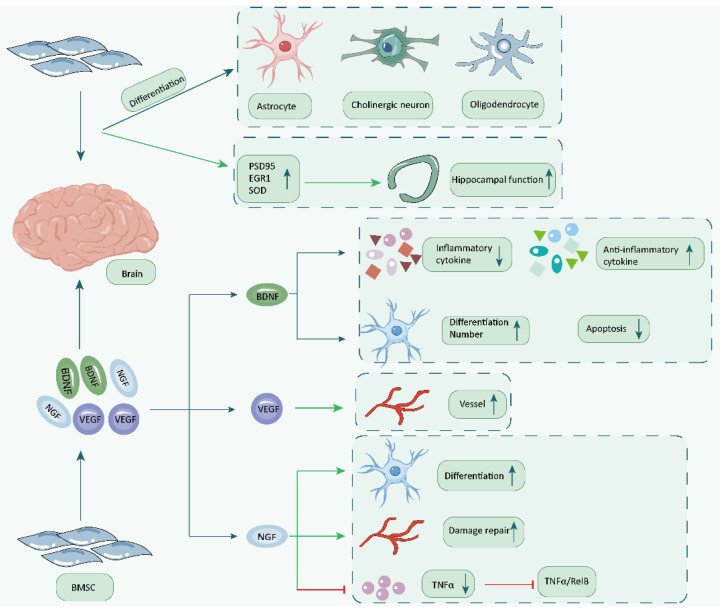
**BMSCs participate in brain regulation through autologous transplantation, secretion of trophic factors, and inhibition of inflammatory factors.** Transplanted BMSCs exhibit the capacity to differentiate into astrocytes, choline acetyltransferase neurons, and oligodendrocytes, thereby contributing to the regulation of brain function. Concurrently, BMSC transplantation enhances superoxide dismutase activity in the hippocampus by upregulating PSD95 and Egr1 expression, consequently ameliorating hippocampal cognitive function. Furthermore, BMSCs play a pivotal role in brain regulation through the action of BDNF, VEGF, and NGF. BDNF, firstly, suppresses the expression of pro-inflammatory cytokines (TNF-α, IFN-γ, IL-1β, IL-6) and promotes the expression of anti-inflammatory cytokines (IL-4, IL-10, TGF-β, IL-11). Additionally, BDNF mitigates neuronal apoptosis and fosters neuronal differentiation and proliferation. Secondly, VEGF orchestrates brain capillary production. Moreover, NGF facilitates neural-like cell differentiation, participates in the restoration of injured brain microvascular cells, downregulates the pro-inflammatory cytokine TNF-α, and inhibits the TNF-α/RelB pathway to mitigate inflammatory responses. The green arrow indicates promotion, while the red arrow indicates inhibition.

BMSCs transplantation can improve cognitive function in aging and diabetic mice, as well as the learning and memory ability of dementia mice. Additionally, BMSCs can differentiate into neurons, astrocytes, and oligodendrocytes, promoting brain function recovery. Furthermore, BMSC secretion and regulation of neurotrophic factors play an active role in brain function recovery. BDNF delivered by BMSCs can effectively alleviate brain function damage, and BMSCs can also secrete BDNF to protect neurons from glutamate toxicity. Moreover, the secretion of NGF by BMSCs can reduce inflammation and improve nerve function. BMSCs can also promote brain angiogenesis, enhance cerebral blood perfusion, and promote brain function recovery through the secretion and expression of VEGF (Figure 2).

#### 3.1.2. BMSCs-Derived Exosomes Regulate Brain Function

EXOs are small vesicles with a diameter ranging from 40 to 100 nanometers, secreted by various cells [53]. They can carry a variety of functional biomolecules, including proteins, microRNAs, and lipids, and play an essential role in intercellular communication [54]. Bone-derived exosomes (BDEs) exhibit multiple neuroprotective effects, such as restoring brain function by promoting angiogenesis, neurogenesis [55], and attenuating neuroinflammation [56]. Additionally, they serve as endogenous carriers for therapeutic agents, such as facilitating astrocyte repair, exerting antioxidant effects, and reducing neuroinflammation via the miR-17-92 cluster [57]. Notably, BDEs can non-invasively cross the BBB, avoiding surgical trauma while stimulating neuronal regeneration and counteracting oxidative damage [58,59]. Specifically, BMSC-derived exosomes enhance angiogenesis, neurogenesis [60], and suppress inflammation [61], thereby facilitating functional brain recovery. They also function as natural delivery vehicles for therapeutic miRNAs [62,63], such as miR-146a (promoting astrocyte repair [64]) and miR-23b (exerting antioxidant effects [65]).

##### BMSCs-EXOs Regulate Brain Function by Regulating Neurons, Microvessels, and Intracerebral Factors

BMSCs-EXOs ameliorated TBI in mice by regulating angiogenesis and neurogenesis, and reducing neuroinflammation [16]. Liu et al. [55] discovered that BMSCs-EXOs, in conjunction with hyaluronic acid-collagen hydrogel, synergistically promoted the differentiation of neural stem cells into neurons and oligodendrocytes, inhibited astrocyte differentiation, induced angiogenesis and neurogenesis (including axon and myelin regeneration, and synapse formation), and even facilitated brain structural remodeling, ultimately enhancing neurological function recovery after TBI. Zhang et al. [57] also demonstrated that BMSCs-EXOs therapy promoted angiogenesis and neurogenesis, reduced hippocampal neuron loss in TBI, and ameliorated sensorimotor and cognitive dysfunction associated with brain trauma. Similarly, Zhuang et al. [66] found that BMSCs-EXOs significantly reduced cortical lesion volume and enhanced cognitive function post-TBI. Moreover, intravenous injection of BMSCs-EXOs could penetrate the BBB and reach the peri-lesion cortex, leading to a significant reduction in glutamate levels and an increase in glutamate transporter one levels in the peri-lesion cortex in TBI mice. This process down-regulated the phosphorylation of P38 MAPK, the levels of pro-apoptotic markers (including cleaved caspase-3 and caspase-9). It increased the number of glutamate transporter one positive cells, ultimately reducing apoptotic neurons around the lesion cortex [67]. Beyond TBI treatment, BMSCs-EXOs also significantly ameliorated various other brain injury-related diseases. BMSCs-EXOs may alleviate neurological function and pathological manifestations in MCAO mice by promoting brain microvascular endothelial cell proliferation and migration [68], attenuating neuronal apoptosis, and reducing inflammatory factors [56]. Following SAH, BMSCs-EXOs improved neurological function, reduced brain water content, maintained BBB integrity [69], inhibited nuclear factor kappa B (NF-κB), activated AMP-activated protein kinase, reduced inflammation, and protected nerves [70]. Duan et al. [67] found that BMSCs-EXOs rich in miR-146a-5p played a neuroprotective and improved function after intracerebral hemorrhage (ICH) by reducing neuronal apoptosis and down-regulating interleukin-1 receptor-associated kinase1 (IRAK1) and nuclear factor of activated T cells 5 (NFAT5) expression. Remarkably, BMSCs-EXOs could ameliorate the pathogenic features of PD by reshaping the inflammatory microenvironment of the substantia nigra and repairing dopamine nerve damage during dopaminergic neuron differentiation, such as reversing the rotational behavior and climbing speed of PD mice [71]. Additionally, lateral ventricle injection of BMSCs-EXOs improved the behavior of mice with sporadic AD induced by streptozotocin (STZ), as evidenced by an increased preference index in field and new object recognition experiments. The injection also inhibited the overactivation of microglia and astrocytes within the hippocampus of AD mice, while decreasing the expression of IL-1β, IL-6, TNF-α, and amyloid-β (Aβ)1-42, and increasing the expression of synapse-associated proteins and BDNF [72]. In terms of ameliorating hippocampal nerve damage, both BMSCs and BMSCs-EXOs up-regulated myelinating factors (myelin%, oligodendrocyte transcription factor2, and opalin), neurotrophic factors (BDNF, fibroblast growth factor2 (FGF2)), synaptic factor (synaptic element), and the expression of chemokine receptor (Cx3cr1) to reduce hippocampal nerve degeneration and demyelination. This successfully restored cognitive and behavioral abnormalities induced by Adriamycin, eliminating Adriamycin-induced chemotherapy-related brain inflammation, apoptosis, and astrocyte and microglia activation. Moreover, antioxidant levels, including glutathione, GSH peroxidase, and SOD activity, significantly increased [73]. BMSCs-EXOs from mice with ischemic brain injury may alleviate ischemic brain injury by inhibiting neuronal death induced by hypoxia and glucose deprivation in astrocytes through the IL-33/ST2 signaling pathway [74].

##### BMSCs-EXOs Regulate Brain Function by Regulating Inflammatory and Oxidative Stress

In addition to promoting the functional recovery of brain injuries by regulating the generation and apoptosis of neurons, the formation of microvessels in the brain, and the expression of various factors, BMSCs-EXOs can also participate in regulating brain function by modulating inflammatory responses and oxidative stress. Wen et al. [75] Conducted in vitro co-culture experiments of BV2 microglia with BMSCs or BMSCs-EXOs and found that BMSCs-EXOs could promote the polarization of activated BV2 microglia, causing them to adopt an anti-inflammatory phenotype, inhibiting the expression of pro-inflammatory cytokines, and increasing the expression of anti-inflammatory cytokines. Tail vein injection of BMSCs-EXOs can reduce cortical cell apoptosis, inhibit neuroinflammation, and promote the transformation of microglia into an anti-inflammatory phenotype in TBI mice. BMSCs-EXOs can also regulate the polarization of microglia/macrophages by down-regulating inducible nitric oxide synthase (iNOS) and up-regulating a cluster of differentiation (CD) 206 and arginase-1 to alleviate the early inflammatory response in TBI, reduce neuropathy, and improve neurobehavioral performance, such as the modified neurological severity score and rotary-rod test. Similarly, Yang et al. [76] found that the proliferation of BV2 microglia in mice exposed to oxygen-glucose deprivation/reperfusion (OGD/R) was accelerated when co-cultured with BMSCs. IL-1β, IL-6, and TNF-α were down-regulated, accompanied by increased levels of the markers of oxidative stress, SOD and malondialdehyde (MDA) [77]. Additionally, BMSCs-EXOs, containing zinc finger antisense 1, also reduced oxidative stress, apoptosis, and inflammation in BV2 cells and MCAO-induced oxidative stress, cerebral infarction, and inflammation in mice. Liu et al. [72] administered BMSCs-EXOs into the lateral ventricle and inhibited the excessive activation of microglia and astrocytes in the AD model mice hippocampus, simultaneously reducing the level of IL-1β, IL-6, TNF-α, synaptic-related proteins, and increasing BDNF protein levels. Li et al. [71] injected BMSCs-EXOs into the PD mice striatum and found that the levels of IL-6, IL-1β, TNF-α, and active oxygen proteins in the PD mice nigra were reduced. HBMSCs-EXOs can be transferred into human microglia, inhibiting the transcription of inflammation-related genes by inhibiting the P38MAPK/P65NF-κB signaling cascade, regulating the polarization and inflammatory response of human microglia, inhibiting the microglial activation, and reducing the pathological changes in the location of the lesion. This prevents the aggravation of the inflammatory response, which is significant in avoiding the subsequent neurological deficits caused by secondary brain injury [78]. Additionally, treatment with BMSCs-EXOs, similar to injection of BMSCs, could restore diabetes-induced cognitive impairment and histological abnormalities. However, it is worth noting that the injected EXOs can be internalized into astrocytes and neurons using fluorescent labeling, but without increasing the number of neurons [23]. These results suggest that BMSCs-EXO exerts neuroprotective and cognitive effects, at least in part, by regulating inflammatory response and oxidative stress.

##### BMSCs-EXOs Regulate Brain Function by Secreting microRNA

The microRNAs (miRNAs) secreted by BMSCs-EXO play an essential role in regulating brain function through various mechanisms. MiRNA-129-5p in BMSCs-EXO inhibits the anti-inflammatory and anti-apoptotic effects of the high-mobility group box one protein (HMGB1)-Toll-like receptor-4 (TLR4) pathway, reducing the levels of HMGB1 mRNA, TNF-α, HMGB1, TLR4, and pro-apoptotic factor p53, thereby alleviating early brain injury after SAH [69]. Overexpression of miR-138-5p promotes the proliferation of astrocytes damaged by OGD, inhibits apoptosis, reduces the expression of inflammatory factors, and ultimately alleviates neuronal damage in IS mice [79]. In vitro experiments to improve the AD model mice show that BMSCs-EXO miR-146a reduces the level of NF-κB and promotes the recovery of astrocyte functions, such as synaptogenesis and correction of cognitive dysfunction [80]. BMSCs-EXO, through negative regulation of caspase-8-dependent apoptotic pathways, inhibits the secretion of miR-134, thereby reducing oligodendrocyte apoptosis in mice [81]. Hu et al. [65] found that BMSCs-EXO miR-23b exhibits an antioxidant effect by inhibiting the phosphatase gene (PTEN) and reducing apoptosis mediated by the NLRP3 inflammasome, promoting the recovery of neurological function in mice with intracerebral hemorrhage. Furthermore, BMSCs-EXO may activate the IL-10/STAT3 pathway by up-regulating miR-181b to inhibit neuroinflammation after TBI [75]. Similarly, treatment with miRNA in exosomes reduces nerve inflammation and cell loss, enhances angiogenesis and neurogenesis, and significantly improves TBI functional recovery in mice [57]. Additionally, studies have shown that restoring miR-124-3p in BMSCs-EXO can enhance the neurological function of neonatal mice with hypoxic–ischemic brain damage (HIBD), reducing the pathological and structural damage of neurons and inhibiting oxidative stress, thereby reducing neuronal apoptosis [82]. For example, BMSCs-EXO miR-150-5p has a protective effect on cerebral ischemia/reperfusion (I/R) injury by inhibiting TLR5 [56]. This research highlights the role of different microRNAs in BMSCs-EXO in regulating brain function, providing valuable insights into the treatment of brain-related diseases and the mitigation of brain damage.

In conclusion, BMSCs-EXOs can enhance brain repair mechanisms by regulating neuronal proliferation and apoptosis, promoting cerebral angiogenesis, and reducing inflammation. Furthermore, BMSCs-EXOs containing different miRNAs exhibit varying effects on brain function. However, exosome-based therapies face critical challenges. Rapid administration (particularly for cancer vaccines or allogeneic cell therapies) requires large-scale exosome production within a short timeframe. Nevertheless, cell culture expansion is constrained by growth kinetics and limited cell yields [83]. Further complications arise from complex isolation protocols, the lack of standardized separation/storage/purification methods, and insufficient clinical translation. Currently, no clinical trials have investigated BMSC-derived exosomes in AD intervention. Current research predominantly relies on murine or cell models, which lack cross-disease comparisons. While BMSC-based therapies show promise, further substantial advancements are imperative.

**Figure 3:**
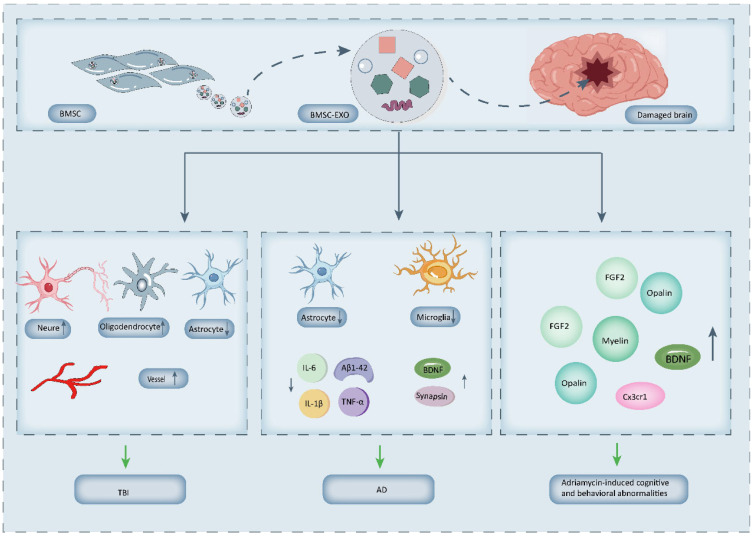
**BMSCs-derived exosomes regulate brain function.** BMSCs-EXO exerts exobiological effects by promoting neural stem cell differentiation into neurons and oligodendrocytes, while concurrently inhibiting astrocyte differentiation. They also suppress NF-kB activity, activate AMPK, mitigate inflammation, modulate blood vessel formation, and enhance mouse function. BMSCs mitigate cortical injury after TBI and ameliorate cognitive function. Moreover, BMSCs-EXO mitigate excessive microglial and astrocytic activation in the hippocampus of AD model mice, resulting in decreased expression of inflammatory markers (IL-6, IL-1β, TNF-α, and Aβ 1-42 protein), inhibition of Tau phosphorylation, modulation of synaptic proteins, and upregulation of BDNF levels, thereby improving AD-like behaviors in mice. Additionally, BMSCs-EXOs upregulate nerve myelination factors (myelin basic protein, Olig2, and oligodendrocyte progenitor cells), neurotrophic growth factors (BDNF, FGF2), synaptic proteins (synaptophysin), and chemokine receptors (Cx3cr1), thereby mitigating hippocampal neurodegeneration and nerve demyelination, successfully restoring cognitive and behavioral abnormalities induced by Adriamycin. The green arrow indicates promotion.

In short, following BMSCs transplantation, these cells migrate to sites of nerve injury or neurodegeneration, where they facilitate nerve tissue repair and restore central and peripheral nervous system function by differentiating into astrocytes and neurons [22]. BMSCs secrete factors such as BDNF, NGF, and VEGF, which protect neurons, enhance neuronal differentiation, mitigate inflammation, and stimulate microvascular formation [34,48]. Although BMSCs-EXO cannot directly differentiate into neurons, they promote neural stem cell differentiation into neurons and oligodendrocytes while suppressing astrocyte formation [55]. This modulation of neuronal cell populations reduces inflammatory responses and oxidative stress [72,75]. BMSCs-EXO also regulate molecular expression in the brain through their cargo, significantly influencing brain function. For example, they attenuate inflammation by inhibiting NF-κB activity, activating AMPK, modulating vascular formation, and improving brain function in mice [70]. Additionally, BMSCs-EXO counteract neurodegeneration and hippocampal demyelination by upregulating neurotrophic factors (such as myelin basic protein, Olig2), oligodendrocyte progenitor cells, growth factors (such as BDNF, FGF2), synaptophysin, and Cx3cr1 [73] (Figure 3).

#### 3.1.3. The Effects and Mechanisms of BMSCs on AD

Multiple studies have demonstrated that BMSCs can ameliorate AD symptoms through the following mechanisms.

First, injection into the lateral ventricle of extracellular vesicles derived from BMSC–EXOs improves behavioral manifestations similar to those in AD by suppressing the overactivation of hippocampal microglia and astrocytes in AD model mice. This leads to reduced expression of IL-1β, IL-6, TNF-α, and Aβ1-42 proteins, inhibition of Tau phosphorylation, and upregulation of synaptic-associated proteins and BDNF [72].

Second, BMSCs transplantation inhibits neuronal apoptosis, enhances hippocampal superoxide dismutase activity, and significantly increases hippocampal BDNF protein levels [15]. BMSC-EXO-derived miR-146a reduces NF-κB levels [80], while BMSC-EXO-derived growth differentiation factor 15 alleviates SH-SY5Y cell damage in an AD model via the AKT/GSK-3β/β-catenin pathway [84].

Additionally, BMSCs combined with other pharmacological treatments can alleviate AD symptoms. For example, breviscapine combined with BMSC therapy activates NF-κB to promote ubiquitin carboxyl-terminal hydrolase L1 expression, reduces Aβ deposition in AD rats, and facilitates the degradation of amyloid precursor protein and β-site APP-cleaving enzyme 1 [85]. Fasudil combined with BMSCs improves cognitive function in AD mice by modulating the peripheral immune system, including downregulating ROCK-II expression and increasing the proportion of anti-inflammatory M2 monocyte phenotypes and phagocytic macrophages in the spleen [86].

In conclusion, BMSCs mitigate AD symptoms through multiple mechanisms. Furthermore, we have summarized the therapeutic applications and mechanisms of BMSCs in brain diseases (Table 1).

### 3.2. Osteoblasts Regulate Brain Function

**Figure 4:**
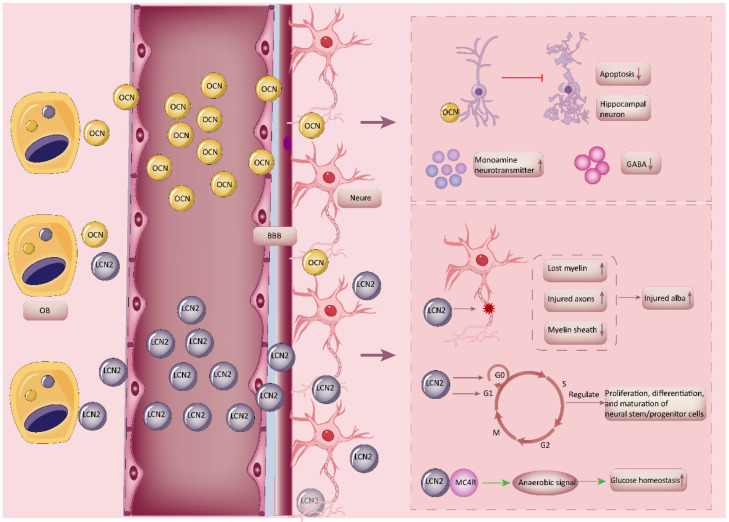
**Osteoblasts regulate brain function.** OBs regulate brain function via OCN and LCN2. OCN crosses the BBB, specifically binds neurons, prevents apoptosis of hippocampal neurons, promotes synthesis of all monoamine neurotransmitters, and inhibits GABA synthesis. LCN2 significantly exacerbated myelin loss, axonal damage, inhibited myelination, and exacerbated white matter damage. LCN2 is involved in the regulation of proliferation, differentiation, and maturation of neural stem/progenitor cells by regulating the G0/G1 cell cycle of neural stem cells. LCN2, by combining with the hypothalamus MC4R, activates the MC4R, which relies on anaerobic signals to maintain glucose homeostasis. The green arrow indicates promotion.

OBs are bone-forming cells of the bone remodeling unit that are essential for bone growth and maintenance [87]. They also have a secretory function and can secrete various factors involved in regulating brain function, such as osteocalcin (OCN) and lipocalin-2 (LCN2) (Figure 4).

#### 3.2.1. OBs-Derived OCN Regulates Brain Function

OCN is a bone-derived endocrine hormone that plays a regulatory role in various biological processes, including those occurring in the brain and bones [88]. It is one of the most abundant proteins in bone and is stably expressed in OBs during maturation [89]. OCN comprises 46 to 55 amino acids and serves as a bone-derived factor with critical regulatory functions. There are several forms of OCN in circulation, including fully carboxylated osteocalcin (cOCN), fully uncarboxylated osteocalcin (ucOCN), and intermediate monocarboxylated and decarboxylated OCN, as well as several cleavage products [90]. COCN is primarily deposited in the bone matrix as a structural component, while ucOCN plays a regulatory role in several organs beyond bone through blood circulation.

Evidence indicates that ucOCN crosses the BBB and accumulates in specific regions of the brain [91,92], primarily the midbrain and brainstem [91]. It crosses the BBB and selectively binds to neurons in the brainstem, midbrain, and hippocampus, demonstrating region-specific binding affinity [91]. For instance, OCN protein colocalizes with GPR158 in the dentate gyrus and CA3 subfield of the hippocampus. The binding of OCN in the DG appears to enhance the hippocampus’ capacity to form discriminative memories, while its binding in the CA3a subregion facilitates memory retrieval [93]. In contrast, the cOCN form passes through the BBB less efficiently [92]. OCN has been shown to improve cognitive function by regulating neurotransmitter synthesis and promoting hippocampal development [91]. OCN has been demonstrated to traverse the BBB and interact with various neurons, including serotonergic neurons located within the raphe nucleus of the brainstem, dopaminergic neurons residing in the ventral tegmental area of the midbrain, and neurons situated within the CA3 region of the hippocampus for explicit binding. This interaction prevents hippocampal neuronal apoptosis, promotes the synthesis of all monoamine neurotransmitters, and inhibits the synthesis of γ-aminobutyric acid (GABA) [91]. Adult OCN^−/−^ mice exhibit passive behavioral phenotypes and several abnormalities in brain structure, neurotransmitter levels, learning and memory, anxiety, and depression phenotypes [94,95]. Dissection revealed a smaller brain size in OCN^−/−^ mice, a 30% reduction in the hippocampal dentate gyrus, and frequent loss of the corpus callosum, both structural alterations consistent with reduced spatial learning and memory abilities. At the biochemical level, OCN^−/−^ mice exhibited significantly reduced levels of monoamine neurotransmitters, including dopamine, serotonin, and norepinephrine, in the midbrain and brainstem. The accumulation of the inhibitory neurotransmitter GABA was also significantly higher in the same regions. These findings are associated with anxiety and depression phenotypes and can be alleviated by intraventricular infusion of OCN in OCN^−/−^ mice [91]. Additionally, the presence of OCN was also found to be associated with age. Khrimian et al. [96] demonstrated that the peripheral delivery of OCN was sufficient to improve memory and reduce anxiety-like behavior in 16-month-old mice after plasma injection from young mice into old mice. Furthermore, the study determined that OCN regulates its effect on cognitive function through interaction with the class C orphan nuclear GPCR Gpr158. However, clinical studies indicate that AD patients with poorer cognitive function exhibit elevated OCN levels, and both plasma and cerebrospinal fluid OCN levels are significantly correlated with cerebral Aβ deposition, tau hyperphosphorylation (pTau), neurodegeneration, and cognitive decline [97]. There are contradictions between clinical studies and basic animal studies, which may be related to differences in anatomical structure. Therefore, further investigation of the role of OCN in bone and brain is needed.

#### 3.2.2. OBs-Derived LCN2 Regulates Brain Function

LCN2 is a typical small secretory protein with a hydrophobic ligand-binding pocket [98]. LCN2 and its receptors are involved in a wide range of physiological processes, including defense against specific bacterial infections [99], regulation of mammalian iron homeostasis, chemotaxis, cell migration, cell differentiation, energy metabolism, and anti- and pro-apoptotic signaling, as well as anti- and pro-inflammatory responses [100]. Initially considered a hormone secreted by adipose tissue, recent studies have shown that LCN2 levels are at least tenfold higher in OBs than in white adipose tissue or other organs. Consequently, bone tissue is the primary organ for LCN2 expression, with OBs being the main source of secretion [101].

LCN2 knockout (KO) mice were injected with peripheral LCN2, demonstrating that LCN2 could traverse the BBB and accumulate in the brain, substantiating its role beyond peripheral tissues [100]. Intravenous injection of radiolabeled human LCN2 into primates revealed that LCN2 crossed the BBB and accumulated in the hypothalamus [102]. Furthermore, LCN2 accumulates in the brain of LCN2-KO mice following intraperitoneal injection [101], providing further evidence of peripherally produced LCN2 entering and accumulating in the brain. Moreover, LCN2 has been shown to disrupt the BBB and exacerbate BBB destruction in mice with ischemic and hemorrhagic stroke [103]. Improved blood–brain barrier leakage and attenuated post-stroke LCN2 induction were observed in stroke-reperfusion-injured mice following specific neutralization of LCN2 with a monoclonal antibody [104]. Furthermore, LCN2 has been implicated in kainic acid-induced BBB leakage in the hippocampus [105]. Thus, LCN2 can enter the brain and accumulate, disrupting the BBB.

White matter, mainly composed of myelin, is essential for cognitive function, and white matter damage contributes to cognitive decline and dementia observed in brain disease [106]. LCN2 can significantly exacerbate myelin loss in mice with MS, spinal cord injury, and stroke [103]. Current research on the effects of LCN2 on myelin primarily focuses on MS. The role of LCN2 in MS-associated myelin pathology may be attributed to its regulation of iron availability and cellular iron uptake. During myelin and oligodendrocyte damage in MS, iron is released into the extracellular space, where a portion undergoes conversion to potentially toxic ferrous iron [107]. In the presence of oxygen-free radicals generated by oxidative bursts, ferrous iron may exacerbate oxidative damage [108], which could be particularly critical in active demyelinating lesions [109]. Additionally, LCN2 may have exacerbated axonal injury in SAH mice through the loss of protective myelin [110]. Furthermore, myelin formation in glial co-culture was inhibited by LCN2 [111]. In conclusion, the evidence suggests that LCN2 can exacerbate white matter damage in brain diseases.

Normal physiological levels of LCN2 also influence adult mouse neurogenesis. Firstly, the cell cycle of neural stem cells in the G0/G1 phase in LCN2-deficient mice is arrested, affecting their proliferation, differentiation, and maturation [112]. Secondly, the expression of LCN2 in the basal hippocampus of neuronal growth regulator 1-deficient mice is significantly lower than that in wild-type mice. However, damage to hippocampal neurons in these mice is mitigated after LCN2 injection [113]. Furthermore, conditioned medium from LCN2-treated glial cells protects neurons and induces synaptic binding proteins and synaptic vesicle proteins [114]. Berard et al. [115] showed that inducing EAE in LCN2-null mice increased disease severity and pro-inflammatory responses, suggesting a neuroprotective role for LCN2. Apart from its role in neurogenesis, LCN2 has been implicated in increased neuronal cell death [116]. In vitro studies have demonstrated that elevated LCN2 expression and secretion can sensitize cells to death, stimulate cell migration, and lead to astrocyte morphological changes [117]. LCN2-induced cytotoxic sensitization is related to iron metabolism and BCL2 and involves astrocytes, activated microglia, and neurons [116].

Although LCN2 is not expressed in the hypothalamus, it has been demonstrated to regulate food intake by traversing the BBB and directly stimulating cAMP signals within the hypothalamus. OB-derived LCN2 binds to the melanocortin four receptor (MC4R) within the hypothalamus, activates MC4R-dependent anorexigenic signaling, inhibits food intake, modulates glucose tolerance and homeostasis, insulin sensitivity, and secretion [101]. Consistent with this, Petropoulou et al. [102] found that LCN2 crosses the BBB of monkeys and binds to the hypothalamus, a brain center that regulates appetite and energy balance. Additionally, in the absence of LCN2, there is a reduction in the number and size of pancreatic islets, as well as the mass and proliferation of beta cells [118]. Thus, LCN2 suppresses appetite by acting on the hypothalamus and affecting islet function.

Several studies suggest that LCN2 deficiency and excess may be associated with depression [119]. A significant elevation in LCN2 levels in the hippocampus has been observed in patients diagnosed with attention deficit disorder who also meet the criteria for depression [120]. In addition, LCN2 KO mice exhibit anxiety-like behavior without any additional intervention [121]. Interestingly, while young LCN2-KO mice (aged between two and three months) showed anxiety-like behavior under control conditions [122,123], no anxiety-like behavior was observed in aged LCN2-KO mice (twelve months old). This finding suggests the possibility that the behavioral differences observed in LCN2-KO mice may undergo a process of normalization as the animals reach maturity [124]. In the CNS, LCN2 is closely associated with altered brain homeostasis and specific regions affected in neurodegenerative diseases, including AD and MS, such as elevated levels of LCN2 in plasma and cerebrospinal fluid (CSF) in patients with mild cognitive impairment [125], depression [126], and MS [127]. This may be related to the increased expression of LCN2 in the whole body [122] and CNS with age [128]. LCN2 has been proposed as a potential clinical biological marker for MS and age-related cognitive impairment [100]. Additionally, the presence of elevated levels of LCN2 in patients with Mild Cognitive Impairment (MCI) is regarded as a transitional state between normal and mild dementia, reflecting not only the overall inflammation of the CNS but also an essential marker of progression from one form of dementia to another [125]. Consequently, elevated levels of LCN2 may be associated with a higher risk of developing AD [125]. Naude et al. [129] demonstrated that LCN2 is present in brain regions associated with AD pathology and mediates β-amyloid cytotoxicity.

Furthermore, LCN2 contributes to elevated risk factors and the development of age-related brain disorders through a variety of mechanisms, including iron metabolism, inflammation, and cell death/survival signaling [100]. LCN2 has a high affinity for binding and transporting iron [130]. Devireddy et al. [130] demonstrated that LCN2 regulates iron homeostasis and can mediate the import and export of iron in cells. Dysregulation and accumulation of iron are observed in various neurodegenerative diseases and CNS injury types and may significantly contribute to CNS damage. For instance, free iron can elevate oxidative stress (via the Fenton reaction), trigger iron-mediated apoptosis, and promote the aggregation of pathogenic proteins such as Aβ [131]. LCN2-mediated cellular iron deprivation induces the interaction of the pro-apoptotic protein BCL2 in various cell types, including astrocytes, neurons, and hematopoietic cell types, resulting in apoptotic cell death [117,130]. Additionally, LCN2 may promote cell death in specific cell types by inducing cellular iron accumulation [132,133]. Unintervened normal LCN2-KO mice exhibit intracellular iron accumulation in particular cell types, including macrophages, hippocampal neurons, and neural stem cells [112,124,134]. Moreover, iron accumulation associated with LCN2 has been observed in several central diseases. Dekens et al. [124] proved that the accumulation of iron in the hippocampus increases (especially in plaque, hippocampal cone, and granule neurons) in transgenic AD mice. At the same time, LCN2 deficiency significantly reduced the accumulation of AD-related hippocampal iron. Ni et al. [132] reported that LCN2 KO mice exhibited lower up-regulation of ferritin during ICH, and LCN2 deficiency resulted in smaller iron-induced lesions, BBB disruption, and less brain swelling. Hochmeister et al. [135] noted that the increase in LCN2 levels in the brain of IS mice paralleled the accumulation of cellular iron, primarily occurring in macrophages/microglia. CSF LCN2 levels in clinically stable MS patients correlated with CSF transferrin levels and basal ganglia iron accumulation [136]. Additionally, intracellular iron accumulation may be an essential mechanism of LCN2-mediated dopaminergic neuron loss [137]. Consequently, LCN2-mediated iron dysregulation (increase or deprivation) can severely affect cell health and viability [112,130,137]. Additionally, LCN2 stimulates oxidative stress responses through iron accumulation. Active iron accumulates in neural stem cells of LCN2-KO mice, resulting in increased oxidative stress [112]. In addition to its regulation of iron metabolism, LCN2 may influence cell viability by stimulating the activation of pro-inflammatory glial cells and secretion of pro-inflammatory cytokines, as well as by inhibiting specific protective pathways, such as signaling through TNF receptor 2 [129].

In conclusion, LCN2 secreted by OBs can traverse the brain through the BBB and play a role in white matter, nerve growth, and neurons. It can also interact with the hypothalamus to affect appetite and contribute to the development of anxiety and depression. Furthermore, LCN2 is implicated in several CNS diseases, including stroke and AD. Its principal mechanism of action may be attributed to the regulation of iron balance, and it is also associated with the inflammatory response and oxidative stress.

#### 3.2.3. The Effects and Mechanisms of Osteoblasts on AD

The effects of osteoblasts on AD are primarily mediated through OCN and LCN2. The role of OCN in AD remains controversial. On one hand, studies have demonstrated that OCN can ameliorate AD pathology through multiple mechanisms: OCN reduces Aβ deposition in the hippocampus and cortex of AD model mice; enhances high γ-band activity in the medial prefrontal cortex; promotes glycolysis in astrocytes and microglia; and inhibits astrocyte proliferation in the hippocampal region [138]. Conversely, clinical studies indicate that AD patients with poorer cognitive function exhibit elevated OCN levels, and both plasma and cerebrospinal fluid OCN levels are significantly correlated with cerebral Aβ deposition, pTau, neurodegeneration, and cognitive decline.

Additionally, suppression of LCN2 has been shown to improve AD symptoms partially [97]. LCN2 deficiency significantly reduces hippocampal iron accumulation associated with Alzheimer’s disease [124], which may alleviate the aggregation of pathogenic proteins such as Aβ caused by elevated iron levels [131]. Furthermore, knocking down miR-96-5p downregulates Foxo1 expression, thereby inhibiting Foxo1-mediated osteoblast-derived LCN2 production. This regulatory cascade ultimately contributes to the alleviation of AD symptoms [139]. The roles of OCN and LCN2 in AD pathogenesis are complex, and further research is required to elucidate their relationship.

### 3.3. Osteocytes Regulate Brain Function

**Figure 5:**
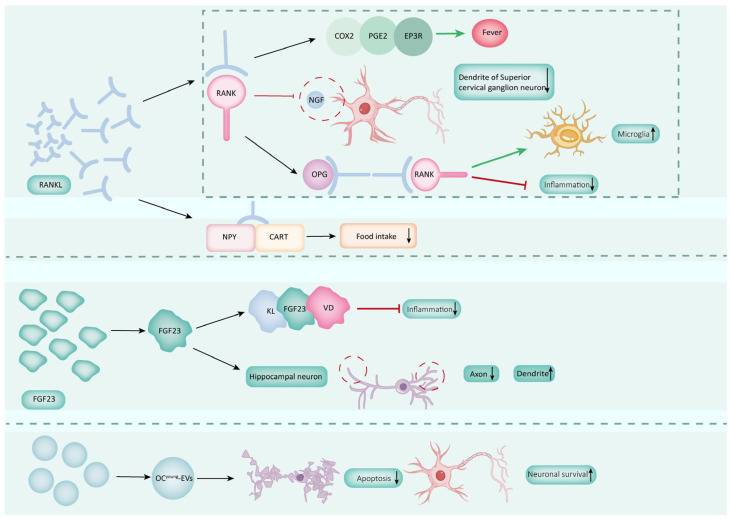
**Osteocytes regulate brain function.** Osteocytes participate in brain regulation through the involvement of RANKL, FGF23, and OC-young-EVs. Firstly, RANKL and RANK combine to activate brain regions involved in thermoregulation and induce fever through the COX2-PGE2/EP3R pathway. Secondly, the RANKL and RANK combine to inhibit the neurite outgrowth of superior cervical ganglion neurons promoted by NGF. RANKL-RANK-OPG then attenuates microglial/macrophage-derived inflammatory responses in ischemic brain tissue while participating in microglial activation. In addition, RANKL could directly reduce food intake in mice through the hypothalamic neuropeptide Y (NPY)/CART pathway. FGF23 also regulates the brain through various mechanisms. FGF23 inhibits inflammatory responses through the KL-FGF23-VD pathway. FGF23 enhances hippocampal function by increasing the number of neurites and reducing dendrites. In addition, OC^young^-EVs reduced cell apoptosis and promoted neuronal survival in APP/PS1 mice. The green arrow indicates promotion, while the red arrow indicates inhibition.

Osteocytes constitute the predominant cell type in bone tissue, dispersed throughout the mineralized bone matrix and forming an interconnected network [140]. They account for approximately 90% to 95% of the total cell count and play a pivotal role in preserving and regulating bone homeostasis. Similarly to neurons, osteocytes possess numerous cilia and cytoplasmic processes, averaging between 40 and 100 processes per cell. Due to their abundance and networked structure, osteocytes are analogous to endocrine cells, releasing secretory factors such as receptor activator of nuclear factor-κB ligand (RANKL), FGF-23, and leptin receptor activator, which regulate bone metabolism as well as kidney and brain function through circulation [141] (Figure 5).

#### 3.3.1. Osteocytes-Derived RANKL Regulates Brain Function

RANKL is a type II homotrimeric transmembrane protein expressed as a membrane-bound and secreted protein. It is generated from the membrane form by proteolytic cleavage or alternative splicing [142]. RANKL is secreted by OBs, osteocytes, hypertrophic chondrocytes, and BMSCs [143]. Osteocytes produce two forms of RANKL: soluble RANKL (sRANKL) and membrane-bound RANKL (mRANKL) [144]. Osteocytes are a significant source of sRANKL [145]. The primary distinction between sRANKL and mRANKL is that mRANKL is bound to the cell membrane and acts through cell–cell contacts. In contrast, sRANKL is derived from the cell membrane of mesenchymal stem cells, chondrocytes, OBs, and osteocytes, requiring cleavage by metalloproteinases produced by OCs for activation [146,147]. Additionally, sRANKL circulates in the bloodstream [148].

RANKL exerts its effects on the hypothalamus, regulating dietary intake and the febrile response. Firstly, RANKL regulates dietary intake. Zhu et al. [149] demonstrated that RANKL can directly reduce food intake in mice through the neuropeptide Y (NPY)/CART pathway mediated in the hypothalamus, leading to weight loss. Furthermore, Enomoto et al. [150] showed that the overexpression of sRANKL reduced food intake and body weight in mice. Elevated sRANKL levels have been observed in patients with anorexia nervosa [151], and RANKL levels correlate with the severity of anorexia nervosa [152]. Secondly, the nuclear factor kappa B (RANK) and RANKL are involved in the hypothalamic febrile response. RANK is a type I trimeric transmembrane protein expressed widely in vivo, such as in mature OCs and dendritic cells [153]. Hanada et al. [154] demonstrated that the RANKL/RANK system functions in the CNS as a critical regulator of fever and basal body temperature in women. The expression of RANK and RANKL is localized to areas of the hypothalamus involved in regulating the febrile response, namely the preoptic area, the middle septal nucleus, and the lateral septal nucleus. Direct injection of RANKL into the lateral ventricle of mouse brains significantly and rapidly increases core body temperature, a response not found in CNS-specific KO RANK mice. RANKL activates brain regions involved in thermoregulation and induces fever through the cyclooxygenase 2 (COX2)-PGE2/EP3R pathway [154]. Furthermore, female mice lacking RANK genes in neurons and astrocytes exhibit elevated basal body temperature, suggesting that the RANKL/RANK system, under the control of sex hormones, also regulates physiological thermoregulation in females [155]. Depletion of RANK, especially in astrocytes, in the mouse CNS attenuates RANKL or LPS-induced febrile effects. This finding suggests that the central RANKL/RANK system mediates the inflammatory febrile response. Prostaglandins serve as essential mediators of fever in the brain, and intracerebral injection of RANKL in mice increased COX2 levels, necessary for prostaglandin synthesis [154]. Central administration of RANKL induces thermogenesis in WT mice [156]. Additionally, RANKL regulates inflammation. RANKL can mitigate the pro-inflammatory effects of Toll-like receptor agonists, suggesting a neuroprotective role in Toll-like receptor-mediated neuroinflammation. Moreover, RANKL can affect nerve growth and development. RANKL and BDNF jointly induce the differentiation of human umbilical cord blood cells into neurons and glial cells, demonstrating a synergistic effect [157]. The binding of RANKL to RANK triggers neurite growth inhibitory signals in developing sympathetic neurons and inhibits neurite growth promoted by NGF in superior cervical ganglion neurons [158].

OPG is a secreted glycoprotein synthesized by various cells, such as OBs, B lymphocytes, and articular chondrocytes [159]. It functions as a decoy receptor for RANKL, which prevents RANKL from interacting with RANK [143]. RANK-RANKL-OPG signaling pathway has been implicated in CNS function and corresponding pathology [160], especially in different areas of nervous tissue damage and repair processes [161], such as in ischemic brain injury. The RANKL-RANK-OPG system plays an essential role in reducing the microglial/macrophage-derived inflammatory response in ischemic brain tissue. After MCAO, RANK, RANKL, and OPG are up-regulated in the cerebral infarction area of mice. By KO OPG or exogenous addition of RANKL, the RANKL/RANK signal may suppress the release of inflammatory cytokines and inhibit further deterioration of infarct volume and cerebral edema [162]. Additionally, several studies have demonstrated that RANKL-RANK-OPG applies to microglia and is mainly involved in microglial activation [163].

In conclusion, RANKL secreted by bone cells regulates brain function through RANKL/RANK and RANK-RANKL-OPG pathways, playing a specific role in regulating dietary intake, the febrile response, and brain injury repair. Additionally, using bi-directional and multivariable Mendelian randomization, researchers found that elevated blood RANKL levels were associated with reduced AD risk [164], suggesting a potential relationship between RANKL and AD.

#### 3.3.2. Osteocytes-Derived FGF23 Regulates Brain Function

FGF23 is an endocrine hormone secreted by osteocytes within the skeleton [165], primarily acting on the kidney and parathyroid to regulate phosphate homeostasis. Although FGF23 is expressed in small amounts in various brain regions, including the cortex, caudate putamen, hippocampus, amygdala, and hypothalamus [166], its levels in the cerebrospinal fluid (CSF) are considerably lower compared to plasma levels. However, in conditions of FGF23 excess, such as in chronic kidney disease (CKD), FGF23 may cross the BBB and potentially disrupt CNS function [167]. This disruption is often manifested as impaired memory formation, a common observation in CKD patients, and is associated with elevated serum FGF23 concentrations [167]. Ursem et al. [168] detected the presence of FGF23 in the hypothalamus but did not find gene expression of FGF23, suggesting that the observed FGF23 protein is not brain-derived, indicating that non-brain-derived FGF23 can enter the brain and exert its effects.

FGF23 is closely linked to various central diseases. Firstly, McGrath et al. [169] demonstrated that higher serum FGF23 levels were correlated with an elevated risk of AD. Drew et al. [167] found significantly upregulated FGF23 levels in patients with cognitive impairment. Secondly, elevated FGF23 levels increase the overall risk of stroke and intracerebral hemorrhage, independent of CKD. Wright et al. [170] discovered an association between elevated FGF23 and cerebral infarction in men. Additionally, the disrupted topology of the left frontal hemisphere network was linked to elevated FGF23, specifically in individuals with cardiovascular risk factors [171]. In CKD patients, FGF23 may impair learning and memory function by acting on the CNS [172,173], potentially affecting the hippocampus. Recent findings by Hensel et al. [172] suggest that FGF23 directly influences neuronal morphology and synaptic density in cultured hippocampal cells, increasing the number of primary neurites while reducing arborization, and enhancing synaptic density in an FGFR-dependent manner, resulting in less complex neuronal morphology. FGF23-deficient mice exhibit dose-dependent cognitive impairment related to the hippocampus. However, no obvious structural or developmental defects in the brain, changes in hippocampal synaptic plasticity, or significant postnatal hippocampal neurogenesis damage were observed [174].

Furthermore, FGF23 is involved in regulating brain tissue through its inflammatory response. Kuro et al. [175] found that the Klotho (KL)-FGF23-vitamin D (VD) axis exerts an anti-inflammatory effect in an inflammatory environment, and the reduction in VD and calcium ions diminishes this neuroprotective effect.

In summary, the effects of FGF23 on the brain primarily manifest in various central diseases, affecting learning, memory, and cognitive function, as well as modulating inflammation in brain tissue through the KL-FGF23-VD axis.

#### 3.3.3. Osteocytes-Derived EVs Regulate Brain Function

Extracellular vesicles (EVs) play an essential role in mediating biological signals between cells [176], often facilitating communication between distant organs [176,177,178]. These vesicles are typically derived from exosomes or plasma membrane microvesicles [179]. Young osteocyte-derived EVs (OCY^young^-EVs), obtained from osteocytes of young mice (2 months old) rather than old mice, have been shown to have a protective effect in AD through the bone-brain axis, leading to improved performance in spatial learning tests in APP/PS1 mice [180]. In the APP/PS1 mouse model, Aβ deposition in the hippocampus is observed at four months, with cognitive impairment evident by six months [181,182]. The mechanisms by which OCY^young^-EVs improve AD pathology in APP/PS1 mice include the reduction in apoptosis in both the hippocampal neuron cell line HT22 and the human AD neuroblastoma cell line SH-SY5Y. Additionally, OCY^young^-EVs ameliorate synaptic deficits and promote neuronal survival in APP/PS1 mice, while also reducing the number of Aβ plaques in brain slices of these mice. These effects are likely due to the combined action of degrading the highly enriched Aβ levels in OCY^young^-EVs and the functional factors related to mitochondrial energy metabolism [180]. Therefore, these studies suggest that EVs secreted by young osteocytes have regulatory effects on the brains of AD patients.

In conclusion, osteocytes secrete FGF23 and EVs to participate in the regulation of central diseases.

#### 3.3.4. The Effects and Mechanisms of Osteocytes on AD

OCY^young^-EVs were demonstrated to reduce neuronal survival by inhibiting apoptosis in both the mouse hippocampal neuronal cell line HT22 and the human AD neuroblastoma cell line SH-SY5Y, while concurrently reducing the quantity of Aβ-labeled plaques and ameliorating AD symptoms [180]. However, studies have shown that osteocyte-derived sclerostin can cross the BBB and exacerbate AD pathogenesis by enhancing Aβ production through the β-catenin-β-secretase one signaling pathway [183]. KL, a transmembrane coreceptor for FGF23, functions as an anti-aging and cognition-enhancing protein that is downregulated in neurodegenerative diseases [184,185]. Cerebrospinal fluid KL levels are reduced in AD patients, while elevated KL concentrations correlate with improved Tau and Aβ biomarker profiles [186]. KL exerts neuroprotective effects by suppressing ROS, thioredoxin-interacting protein, NF-κB activation, NLRP3 inflammasome activity, and neuronal cell death [187]. Both FGF23 knockdown and α-KL overexpression attenuate AD-induced inflammation by activating the Wnt/β-catenin pathway in peripheral blood mononuclear cells, thereby ameliorating AD symptoms [188]. This mechanistic link is supported by evidence that Wnt/β-catenin pathway activation suppresses Aβ production and PTau [189], while concurrently modulating neuroinflammation in AD pathogenesis [190]. In conclusion, osteocytes exert a dual regulatory role in AD progression [191].

### 3.4. BMMs Regulate Brain Function

#### 3.4.1. Transplanted BMMs Regulate Brain Function

BMMs form a diverse cell population derived from bone marrow, comprising variously mature B cells, T cells, monocytes, and a smaller proportion of progenitor cells such as hematopoietic stem cells, mesenchymal stem cells, endothelial progenitor cells, and tiny embryonic-like cells [192]. BMMs play an essential role in regulating brain function, capable of crossing the BBB to reach the ischemic cortex and white matter [193], offering therapeutic benefits for various cerebrovascular diseases. Treatment with BMMs can attenuate inflammation and oxidative stress in the ischemic brain of mice, improve cerebral blood flow, and significantly protect nerves and cerebral ischemic white matter [194]. Long-term BMMs treatment can promote axonal sprouting in mice with cortical ischemia, aiding in the reconnection of damaged neuronal circuits and significantly enhancing motor performance [195]. These findings provide promise for the use of BMMs in treating subcortical ischemic vascular dementia [196]. Additionally, Wang et al. [193] observed that mice treated with BMMs following permanent bilateral occlusion of the common carotid arteries (2VO) exhibited improved learning and memory abilities, increased vascular density, and reduced white matter damage compared to control mice. Furthermore, the protein expression of VEGF, phosphorylated rapidly accelerated fibrosarcoma 1 (Raf1), and extracellular signal-regulated kinases 1 and 2 (ERK1/2) was significantly increased, suggesting an upregulation of the VEGF-VEGFR2 signaling pathway. Wang et al. [197] demonstrated that pretreatment with an adequate dose of VEGF could amplify the therapeutic effects of BMMs transplantation in 2VO mice, improving learning and memory, enhancing vascular proliferation, and reducing neuronal degeneration, possibly by promoting BMMs migration into the ischemic brain. In a study by Kitamura et al. [198] Involving permanent middle cerebral artery occlusion (pMCAO), BMMs with vascular endothelial and microglial/macrophage characteristics were transplanted, leading to significant inhibition of astrocytes and microglia in the damaged part of the mouse brain, improved cerebral blood flow, and enhanced sensorimotor behavioral function compared to the control group. Moreover, inflammatory factors were regulated, with a significant increase in anti-inflammatory cytokines, A2 astrocyte/anti-inflammatory microglial markers, and vascular endothelial markers such as VEGF, and a substantial decrease in pro-inflammatory cytokines and A1 astrocyte/pro-inflammatory microglial markers. Jiang et al. [199] injected BMMs or microglia into the tail vein of mice 24 h after pMCAO and found that BMMs injection significantly reduced infarct volume and brain water content compared to microglia injection. Additionally, BMMs injection reduced neurological deficit scores, indicating that intracerebral delivery of BMMs is an effective cellular therapy for chronic stroke. The CXCR4+ CD45- BMMs subset was found to be superior to non-isolated BMMs in improving brain injury in mice with transient middle cerebral artery occlusion (TMCAO) [200].

Apart from their therapeutic effects on cerebral ischemia, BMMs have also demonstrated a role in treating cerebral hemorrhage. Liem et al. [201] reported that autologous BMMs transplantation could improve motor function and reduce muscle spasms in children with neonatal intracerebral hemorrhage. Additionally, Ogawa et al. [202] found that BMMs transplantation combined with chronic stroke training activated ipsilateral and contralateral gene expression. BMMs have shown promising therapeutic effects in treating TBI. The dichotomous Glasgow score of BMMs in children with severe TBI at 6 months after intravenous infusion indicated that 70% of children had a good outcome, 30% had moderate to severe disability, and all survived. This suggests that intravenous infusion of BMMs is feasible and safe for treating severe TBI in children [203]. Early autologous BMMs intrathecal transplantation and neural rehabilitation intervention for patients with mild TBI younger than 18 and injured for less than five years showed good intervention results in cognition and attention [204]. Additionally, the treatment of TBI in adults has also demonstrated positive effects, with down-regulation of inflammatory biomarkers after autologous BMMs infusion [205]. BMMs can improve cognitive function and address intelligence problems. Kanamaru et al. [206] found that intravenous transplantation of BMMs improved cognitive function and prevented neurodegeneration in two different AD mouse models, DAL101 (DAL) mice and Tg2576 (APP) mice. BMMs transplantation inhibited neuronal loss in DAL mice and restored memory impairment to almost the same level as in WT mice, reducing Aβ deposition in the brains of APP mice. In the treatment of patients with intellectual disabilities, the intervention group receiving intrathecal administration of autologous BMMs and standard neurological rehabilitation had a greater improvement in symptoms than the control group receiving only standard neurological rehabilitation. This effect was specifically significant in the age group of children < 18 years old and patients with mild intellectual disability [207]. The transplantation of BMMs also contributes to neural recovery and has anti-inflammatory effects. Transplantation of BMMs can enhance neuroprotection and nerve regeneration following optic nerve injury, possibly mediated by FGF-2 [208]. BMMs release NGF, which promotes sciatic nerve regeneration in adult mice by stimulating the Schwann cell and satellite cell proliferation, or a combination of both [209]. Moreover, Leal et al. [210] observed that BMMs transplantation promoted neuroprotective and anti-inflammatory effects after status epilepticus in mice. Prado-Lima et al. [211] found that BMMs transplantation restored sucrose preference in chronically stressed mice, alleviated inflammation in both the peripheral and central nervous system, and reduced DNA damage. Additionally, BMMs also regulated the secretion of brain substances; transplantation of BMMs prevented chronic mild stress-induced increases in HMGB-1 expression in the hippocampus and spleen, increased BDNF expression in both tissues, and prevented increases in IL-1β expression in the hippocampus. However, no effect of transplantation was observed on TNF-α expression [212].

In conclusion, the transplantation of BMM has demonstrated promising therapeutic effects in various cerebrovascular diseases and traumatic brain injuries, improving cognition, intelligence, and restoring neurological function.

#### 3.4.2. Preosteoclasts-Derived PDGF-BB Regulates Brain Function

Platelet-derived growth factor BB (PDGF-BB) is an angiogenic factor secreted by tartrate-resistant acid phosphatase-positive (TRAP+) preosteoclasts [118,213,214].

Selective knockout of PDGF-BB from preosteoclasts resulted in an approximately 40% decrease in serum PDGF-BB concentration in mice, indicating that preosteoclasts are a significant source of serum PDGF-BB under healthy conditions [215]. Additionally, TRAP+ preosteoclasts are the primary cell type contributing to elevated serum PDGF-BB during aging and metabolic stress [213]. Moreover, preosteoclasts are an essential source of plasma PDGF-BB, with plasma PDGF-BB exhibiting a more significant age-related increase than serum PDGF-BB [215].

PDGF-BB/PDGF receptor (PDGFR β) signaling mediated pericyte-endothelial crosstalk plays a critical role in embryonic development [216,217,218], coverage of brain microvascular pericyte in adulthood, and the establishment and maintenance of BBB homeostasis [217,219]. Significantly, the continuous increase in PDGF-BB from preosteoclast cells can penetrate the endothelium to reach brain cells and reduce hippocampal pericytes [215]. However, the normal physiological range of PDGF-BB is also crucial for pericyte survival, and loss of PDGFR in the beta cells can lead to pericyte apoptosis [220,221].

Pericytes, which line the walls of brain capillaries and share the same basement membrane as capillary endothelial cells, are key cell types in maintaining the integrity of the BBB [222,223]. Dysfunction and deficiency of brain capillary pericytes lead to BBB disruption, contributing to the pathogenesis of many neurological diseases, including AD, stroke, traumatic brain injury, diabetes mellitus, and amyotrophic lateral sclerosis [218]. In addition to hippocampal pericyte loss, elevated levels of circulating PDGF-BB, produced by preosteoclasts, can lead to hippocampal microvascular damage, BBB disruption, cognitive decline (including impaired working and recognition memory), and cerebrovascular disease. Age-associated pericyte loss and BBB damage phenotypes were reduced by matrix metalloproteinase (MMP) activity inhibition, as increased PDGF-BB ligand-induced upregulation of MMP14. MMP14 in the brain pericyte cell membrane cleaves the extracellular domain of PDGFRβ receptors, resulting in reduced PDGF-BB/PDGFRβ signaling, thereby disrupting the BBB [215]. Normalization of PDGF-BB in the circulation ameliorates age-related hippocampal microvascular damage and cognitive decline [215]. In conclusion, PDGF-BB secreted by preosteoclast cells is the major contributor to the increase in serum PDGF-BB in response to aging and metabolic stress. Additionally, PDGF-BB secreted by preosteoclasts can enter the brain and reach brain cells, affecting cognitive and memory functions.

**Figure 6:**
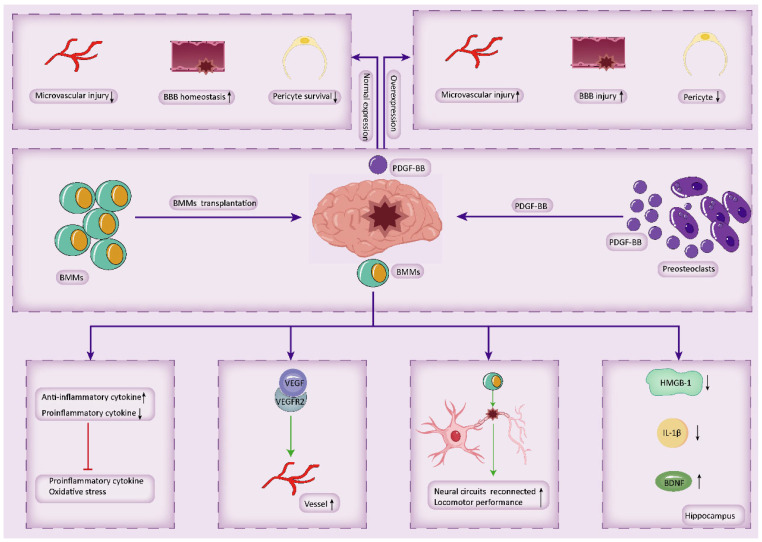
**BMMs regulate brain function.** BMMs transplantation has demonstrated therapeutic effects through a variety of mechanisms. First, it significantly increased anti-inflammatory cytokines while decreasing pro-inflammatory cytokine markers, inhibiting inflammation and oxidative stress in the ischemic brain of mice. Additionally, it upregulated the VEGF-VEGFR2 signaling pathway, promoted angiogenesis, and enhanced cerebral blood flow. Second, BMMs treatment facilitated axonal sprouting, rewiring damaged neuronal circuits, and significantly increased motor performance in mice with cortical ischemia. Moreover, BMMs transplantation prevented CMS-induced increases in HMGB-1 expression in the hippocampus and prevented increases in IL-1β expression in the hippocampus, while increasing BDNF expression in the hippocampus. In addition, the broken bone cells before the regular expression of PDGF-BB can maintain a steady state of the BBB, reduce and promote cell survival, improve microvascular damage to the hippocampus, and excessive expression of hippocampus-induced microvascular damage, BBB damage, and loss of cells in the hippocampus over weeks. The green arrow indicates promotion.

Furthermore, BMMs can improve the symptoms of cerebral hemorrhage and ischemic diseases, and possess neurological recovery and anti-inflammatory functions. Preosteoclasts secrete PDGF-BB to regulate brain functions, including cognitive and memory functions. OCs, as one of the most common cells in bone, have functions in bone resorption and secretion. The regulation of the brain by OCs may occur through the secretion of bone morphogenetic proteins (BMPs) and sphingosine-1-phosphate (S1P) [224]. OCs can secrete various BMPs, including BMP2, BMP4, BMP6, and BMP7 [225]. BMPs may regulate brain function by promoting the fate of astrocytes and inhibiting the differentiation of oligodendrocytes into myeloid cells and neurons [226]. Additionally, OCs can secrete S1P, which regulates brain function by promoting the shift in microglial polarization from the M1 phenotype to the M2 phenotype [227]. However, there is currently no direct evidence showing that BMPs and S1P, which are involved in brain regulation, are secreted by OCs. Furthermore, other factors secreted by OCs, such as collagen triple helix repeat containing 1 [228] and Wnt gene family 10B, have not been identified as mediators of brain function regulation. Further research is needed to clarify the brain regulatory mechanisms of OCs (Figure 6).

#### 3.4.3. The Effects and Mechanisms of Osteoclasts on AD

Currently, there is limited research on the impact of osteoclasts on AD. Intravenous transplantation of BMMs has been shown to ameliorate symptoms in AD model mice by inhibiting neuronal loss and reducing Aβ deposition in the brain [206]. Wu et al. demonstrated that osteoclasts may accelerate AD progression by impairing the immune cell-mediated peripheral clearance of A [191].

## 4. Osteoporosis and AD: A Common Pathophysiology

The interactions between bone and brain form a complex dynamic network through shared pathological mechanisms and molecular signaling pathways. This ‘brain-skeletal axis’ is particularly evident in the comorbidities of neurodegenerative diseases and skeletal metabolic abnormalities. Numerous studies have highlighted the bidirectional and early relationship between cognitive impairment, dementia, fragility fractures, and low BMD [229,230]. The co-existence of AD and osteoporosis is more prevalent than normal cognition and osteoporosis, and participants with AD have an increased likelihood of developing osteoporosis within five years, independent of age, gender, vitamin D levels, and comorbidities [231]. This dynamic cerebral-skeletal axis is mediated by common pathological factors, including inflammation, oxidative stress, and hormonal imbalances that exacerbate neurodegeneration and bone loss. Bone-derived proteins, such as OCN and SOST, affect brain function, while neurodegenerative processes, including Aβ aggregation and pTau, negatively impact bone remodeling [232].

### 4.1. The Pathogenesis Mechanisms of AD

Aβ plaques and pTau NFTs represent the two most prominent molecular hallmarks of AD [233,234].

The pathological aggregation of the tau protein constitutes one of the core pathological mechanisms in AD. Under physiological conditions, the positively charged proline-rich region (PRR) maintains stable tau-microtubule binding through charge repulsion with the positively charged microtubule-binding repeat domain (MTBR). In the disease state, hyperphosphorylation of the PRR (along with other post-translational modifications [PTMs] such as acetylation or ubiquitination) converts its net charge from positive to negative, inducing conformational changes in tau. This transformation facilitates tau detachment from microtubules, resulting in microtubule destabilization [235,236,237]. The dissociated soluble hyperphosphorylated tau (pTau) undergoes abnormal intracellular aggregation, impairing endosomal trafficking and compromising dendritic structure/neuronal function. Simultaneously, liberated tau promotes trans-synaptic propagation via binding to low-density lipoprotein receptor-related protein 1, establishing a “tau pathology network” [237,238]. Subsequently, pTau undergoes further modifications (such as acetylation/ubiquitination of the MTBR), progressively aggregating into semi-soluble pretangle structures (AT8-positive) that ultimately coalesce into insoluble neurofibrillary tangles (NFTs) (AT8/silver stain-positive) [238,239]. Within NFTs, the MTBR fibrillar core is enveloped by a “fuzzy coat” containing hyperphosphorylated PRR and C-terminal domains [239]. During late-stage pathology, proteolytic truncation removes the immunogenic AT8 epitopes from this fuzzy coat, leaving only the silver-stainable MTBR fibrillar core [240].

Amyloid precursor protein (APP) is ubiquitously expressed in the brain, particularly enriched at neuronal synapses, where it participates in neuroprotection, synaptic plasticity, and neuronal development [241]. Its proteolytic product Aβ is secreted extracellularly, undergoing sequential aggregation from oligomers to protofibrils and finally plaques [242]. Aβ aggregates disrupt neuronal communication, trigger neuroinflammatory immune responses, and induce neuronal death. Concurrently, by elevating intracellular calcium levels, Aβ activates calpain-mediated CDK5 hyperactivation, exacerbating pTau. During AD progression, Aβ plaques or pTau accumulation aberrantly activate astrocytes and microglia, which release pro-inflammatory cytokines that accelerate neuronal damage and death. This process is often synergistically driven by intrinsic/extrinsic factors such as oxidative stress [243]. Beyond these core pathways, mitochondrial dysfunction, genetic predisposition, such as familial AD gene mutations, and environmental factors, such as traumatic brain injury, also contribute to AD pathogenesis.

In addition, multi-omics research has also been widely applied to the study of AD and has achieved specific results, providing new perspectives for AD research. Transcriptomic studies of AD have elucidated multi-layered pathological mechanisms. Single-nucleus RNA sequencing (snRNA-seq) analyses revealed that LINGO1, a negative regulator of myelination, is significantly upregulated in AD [244], with abnormal activation of myelination-related pathways observed in both the human prefrontal cortex [244] and entorhinal cortex [245]. Notably, APOE expression in the entorhinal cortex exhibits cell-type-specific dysregulation—suppressed in oligodendrocyte precursor cells and astrocytes but upregulated in microglia—while LINGO1 and NEAT1 are highly expressed in AD subpopulations [245]. Integrated multi-omics studies (snRNA-seq combined with snATAC-seq) further identified cell-specific transcriptional regulatory networks driving AD, such as the “peak-gene-TF” triad involving ZEB1 in neurons and MAFB in microglia [246]. AD risk loci are significantly enriched in microglial enhancer regions (e.g., binding pockets of SPI1, ELF2, and RUNX1), with late-stage AD exhibiting epigenomic erosion that leads to loss of cellular identity [247].

Spatial transcriptomics (10x Visium) elucidated the spatiotemporal dynamics of pathological progression: in both sporadic AD and Down syndrome-associated AD, amyloid plaque-adjacent regions showed activation of genes related to inflammatory responses, lysosomal degradation, and endocytosis, alongside aberrant upregulation of myelination-related genes in oligodendrocytes [248]. Single-cell RNA sequencing (scRNA-seq) further uncovered Trem2-dependent disease-associated microglia (DAM) and a novel reactive oligodendrocyte population marked by Serpina3n/C4b, suggesting impaired neuron-glia metabolic coordination in AD [249]. At the genetic risk level, the intronic variant rs405509 within the APOE promoter region was significantly associated with increased AD risk across multiple populations [250].

### 4.2. The Treatment Mechanisms of AD

Core Molecular Mechanisms of AD Treatment encompass strategies ranging from the clearance of pathogenic protein aggregates and oligomers (Aβ and Tau), modulation of protein phosphorylation via O-GlcNAcylation, and counteracting neuroinflammation and oxidative stress, to the activation of beneficial cellular responses, such as microglial phagocytosis. These approaches collectively aim to mitigate or reverse the neurodegenerative processes associated with AD.

Although Aβ-targeted therapies have not yet achieved permanent remission of AD, they remain a central focus of research. The oral small-molecule prodrug ALZ-801 functions by inhibiting Aβ oligomer formation, particularly in high-risk populations such as APOE4/4 homozygous individuals [243]. Among monoclonal antibody therapies, Lecanemab, an IgG1 antibody, selectively clears soluble Aβ protofibrils predominant in early-stage AD [251], while Donanemab targets insoluble N-terminal pyroglutamate-modified Aβ within amyloid plaques [252].

For the tau protein, another key pathological hallmark of AD, characterized by abnormal aggregation and hyperphosphorylation (pTau), therapeutic strategies focus on disrupting its cascade. IgG1 antibodies targeting the HVPGG motif recognize microtubule-binding domains, inhibiting extracellular Tau seeding and aggregation while reducing Tau deposition in animal models [253,254]. Small-molecule inhibitors such as methylene blue and leuco-methylthioninium bis (hydromethanesulphonate) bind specific cysteine residues (Cys291/322) in Tau, interfering with pathological aggregation [255]. Curcumin has also demonstrated inhibitory effects on tau aggregation in experimental systems [256]. Furthermore, enhancing Tau O-GlcNAcylation counteracts phosphorylation and aggregation. The OGA inhibitor LY3372689, which efficiently crosses the blood–brain barrier and elevates O-GlcNAcylation levels, represents a potential therapeutic approach [243].

Neuroinflammation and oxidative stress pathways are also critical intervention targets. NE3107 suppresses the NF-κB/ERK signaling pathway and reduces MAPK activation, thereby decreasing pro-inflammatory cytokine release, such as TNFα, IL-1α, and alleviating neuroinflammation [257,258]. Ladostigil mitigates oxidative stress (e.g., H_2_O_2_-induced damage) and restores the expression of NADPH oxidase and catalase genes; clinical studies confirm its ability to delay hippocampal atrophy in MCI patients and reduce AD conversion rates in non-APOE4 carriers [254]. Excessive calpain-2 activation is closely linked to pTau formation, and the highly selective inhibitor NA-184 exhibits neuroprotective potential in preclinical studies [259].

Notably, microglial function modulation has emerged as a novel therapeutic direction. AL002c, a TREM2 receptor agonist, enhances microglial phagocytic activity and promotes the encapsulation of Aβ deposits, forming a “protective barrier” [260].

### 4.3. Low Bone Density Exacerbates AD

There is a significant association between low BMD and AD, which can increase the risk of brain-related diseases. Females with lower BMD have a higher risk of developing AD, especially in the femoral neck region [261]. Bone loss in patients with AD is strongly associated with hypothalamic atrophy, and this pathological process precedes structural brain changes [262]. There is also a significant association between fracture history and dementia risk. A 12-year retrospective analysis revealed that individuals with a history of bone fractures were 41% more likely to develop dementia, even after controlling for variables such as age, gender, degree of urbanization, personal illness, and comorbidities [263].

There is a robust association between BMD and brain diseases, with underlying mechanisms involving various signaling pathways and proteins secreted by the bone. mTOR and MAPK pathways have emerged as key molecular targets in bone metabolic disorders by finely regulating the osteogenic-osteoclast balance. In AD studies, it has been found that the MAPK signaling pathway and the mTOR signaling pathway play essential roles in the onset and development of AD. The specific functions of mTOR in the brain include axonal regeneration and ejection, myelin formation, and channel expression [264]. In addition, some proteins secreted by the skeleton, such as OCN and OPN, positively support brain function. For instance, OCN is secreted by osteoblasts and enters the brain to stimulate the production of key neurotransmitters such as dopamine and serotonin, which are essential for memory and emotional balance [92]. However, OCN levels decline with age, which is not only associated with aging but also with cognitive decline and increased susceptibility to Aβ plaques, a typical feature of AD.

### 4.4. AD Exacerbates Bone Loss

AD also impairs bone health to some extent. Firstly, patients with AD have a doubled risk of fragility fractures, while their risk of hip fragility fracture reaches even 2.5 times that of patients without AD [265]. This may be related to the fact that cognitive impairment affects mobility and negatively impacts musculoskeletal biology, leading to lower BMD and increased fracture risk. Secondly, Loskutova et al. [2] found patients with early AD experience a simultaneous decline in bone density, whole brain volume, and limbic gray matter volume, with cognitive decline. However, this association was not observed in non-demented controls. This finding suggests a correlation between alterations in brain structure and bone loss in patients diagnosed with AD.

Aβ aggregation may trigger AD pathology due to the loss of physiological function [266], while promoting osteoclast bone resorption by enhancing NF-κB activity, ERK phosphorylation, and calcium oscillatory signaling pathways, thus becoming a potential target molecule for osteoclast-associated disorders such as OP [267]. In addition, pTau and Aβ plaque formation are intensified by elevated levels of TNF-α and IL-6. These pro-inflammatory factors not only activate microglia in the brain but also promote the production of RANKL in the bone, which in turn stimulates osteoclast development and enhances bone resorption, a characteristic feature of OP [268]. Moreover, PTau also indirectly harms bone by interfering with fundamental systemic pathways such as Insulin-like Growth Factor 1 (IGF-1), which promotes bone development [269].

In conclusion, low BMD and AD interact with each other. Low BMD exacerbates the incidence of AD, and MAPK and mTOR may act as signaling pathways for the two diseases to communicate with each other. Patients with AD also experience bone loss, which may be related to their limited mobility on one hand and the regulation of bone resorption by AD on the other.

## 5. Dual Modulation of Bone and Brain Function by Drugs

### 5.1. Effects of Osteoporosis Medications on Cognitive Functioning

Selective estrogen receptor modulators such as raloxifene exhibit dual roles in brain function, acting as both an estrogen receptor agonist and an antagonist, thereby reducing the risk of estrogen-dependent tumors while having complex effects on cognitive function [270]. Studies have shown that 60 mg/day of raloxifene promotes verbal memory, while a dose of 120 mg/day mitigates the risk of MCI and AD [271]. However, a clinical trial involving female patients with mild to moderate AD found that 120 mg/day of raloxifene had no significant effect on ADAS-Cog scores [272]. In addition, while estrogen enhances learning and memory, it may also trigger cognitive impairment in specific striatum-dependent memory tasks [273]. Bisphosphonates improve cognitive function by inhibiting osteoclast activity and bone resorption, and inhibiting acetylcholinesterase and cholesterol synthesis [274]. Consistent with this, patients with osteoporosis treated with bisphosphonates have a significantly reduced risk of dementia [275] and may have enhanced cognitive function [276]. Research indicates that osteoporotic patients receiving bisphosphonate therapy or estrogen supplementation exhibit risks of developing dementia that are 0.73-fold and 0.76-fold lower, respectively, compared to untreated osteoporotic patients [275]. In hemodialysis patients without a history of stroke or dementia, RANKL levels were positively correlated with short-term memory, mental operations, and abstract thinking [277]. Denosumab, a human monoclonal antibody, reduces osteoclastogenesis and bone resorption by inhibiting the binding of RANKL to RANK while modulating neuroinflammatory responses [278]. Bisphosphonates, such as alendronate and zoledronate, not only increase BMD but also exhibit anxiolytic and antidepressant effects in the treatment of postmenopausal osteoporosis [279].

### 5.2. Neuropharmacological Modulation of Bone Density

On the other hand, neurological drugs can also affect bone density. Cholinesterase inhibitors in elderly AD patients not only improve cognitive function but are also associated with better hip fracture healing and increased BMD [280]. Cholinesterase inhibitors reduce fractures by enhancing gait, balance, attention, and executive function and reducing the risk of falls [281]. In addition, ionotropic glutamate receptor agonists and antagonists play an essential role in osteoblast differentiation and osteoclast formation [282]. As a commonly prescribed AD medication, donepezil has also been shown to downregulate RANK expression in BMMs, thereby inhibiting osteoclast differentiation. This process was accompanied by downregulation of c-Fos and upregulation of inhibitor of DNA binding 2. Furthermore, donepezil administration prevented RANKL-induced bone loss in mice, which was attributed to osteoclast-mediated suppression of bone resorption [283]. These studies suggest that different drugs affect brain function and osteoporosis through multiple mechanisms, providing new perspectives for the integrated treatment of neurodegenerative diseases and osteoporosis.

## 6. Clinical Applications—Whole Body Vibration Therapy to Regulate Bone Brain Crosstalk

Whole-body vibration (WBV) is a physical intervention delivered to the whole body by mechanical vibration, and its mechanisms of action and effects have been widely researched in both the brain and skeletal system. The modulation of brain function by WBV is primarily manifested in three aspects: firstly, WBV stimulates brain regions to enhance neurological function and synaptic plasticity through specific parameters. 27 Hz WBV activates motor networks and the prefrontal cortex in healthy adult males [284]. In addition, intermittent high-frequency WBV (45 Hz, 2 min 30 s training, 2 min 30 s recovery) has a positive effect on synaptic plasticity [285]. Second, WBV is involved in neuroinflammation, suppression, and injury repair. WBV attenuates brain injury by modulating neuroinflammation: vibration training at 40 Hz (twice a day for 30 days) reduced the infarct area and decreased the expression of inflammatory markers such as Caspase-1 and IL-1β in stroke rats [286]; WBV also up-regulated the anti-inflammatory factor IL-10 mRNA, while inhibiting the pro-inflammatory factor TNF-α [287]. In a traumatic brain injury model, WBV improves brain function by modulating neuroinflammation [288]. In addition, WBV regulates neurotrophic factors and neurotransmitters. WBV promotes the expression of BDNF, IGF-1, etc., and enhances neurogenesis [286]. However, the effects of WBV on the brain are dependent on parameter settings: long-term exposure to low-frequency WBV, such as 30 Hz, four hours per day, resulted in memory impairment and brain tissue damage in rats [289].

WBV promotes bone formation and improves bone density. WBV directly modulates bone metabolism through mechanical stimulation: 30 Hz, 15 min/dose of WBV, 8 times/week for 8 weeks, alleviates ovariectomy-induced osteoporosis and inhibits bone resorption [290]. 45 Hz, 15 min/dose of WBV for 5 days/week for 8 weeks improves femoral mechanical strength in type 2 diabetic rats [291]. Clinical evidence suggests that WBV significantly increases lumbar and femoral BMD in postmenopausal women [292].

WBV has a common regulatory mechanism in the brain and bone. On the one hand, WBV (2 min of vibration, 30 s of rest, 12 weeks, 3 times/week, 36 times in total) significantly up-regulated the expression of Fibronectin Type III Domain Containing 5 (FNDC5) in bone, which promoted the proliferation of osteoblasts and reduced the number of osteoclasts [285]. Additionally, the number of Purkinje cells and hippocampal pyramidal neurons was significantly elevated, synaptic plasticity was enhanced, and bone mass increased [285]. On the other hand, increased levels of FNDC5 restore synaptic plasticity and prevent memory impairment in AD patients [293]. Additionally, FNDC5 improved cognitive function in AD model mice by enhancing BDNF-mediated synaptic plasticity [294]. Thus, FNDC5 is a co-interacting molecule in WBV to strengthen the brain and bone. In addition, PGC-1α-induced increases in FNDC5 expression in mouse primary cortex and hippocampal neurons promoted BDNF expression [295]. The PGC-1α/FNDC5/BDNF pathway activates cyclic AMP response element-binding proteins, leading to positive feedback that amplifies its expression, while FNDC5 regulates BDNF production to mediate exercise’s benefits on brain function and health [296].

In conclusion, WBV achieves synergistic interventions on the brain and skeletal system through shared molecular mechanisms, such as the FNDC5/BDNF pathway, and bidirectional regulation of the bone-brain axis. The effects are highly dependent on vibration parameters: high-frequency short-duration training, such as 45 Hz, intermittent, is beneficial for both brain and bone. It may achieve synergistic effects through the FNDC5/BDNF pathway. Additionally, Li et al. analyzed the skeletal improvements induced by different WBV treatment regimens in recent years. They found that the primary parameters employed were 20 to 40 Hz, 2 to 3 times per week, and 5 to 10 min of vibration [292]. Consequently, there is currently no standardized protocol for this therapy in clinical practice. As a non-pharmacological intervention, WBV is widely applied; however, it also presents contraindications, including patients who are at risk of acute thrombosis or embolism, individuals with implanted devices, pregnant women, and those with active tumors or metastatic lesions. Long-term exposure to low frequencies, such as 30 Hz, high intensity, may impair brain function but improve bone metabolism, a trade-off. Future studies should further optimize vibration parameters to balance the synergistic health benefits of brain and bone.

## 7. Future Directions and Challenges

Integrating multi-omics approaches to decipher bone-brain interactions is of great significance. Mone Zaidi et al. [297] explored in depth the regulatory role of bone-secreted factors on various organs with the help of various technological tools, such as RNA-seq, proteomics, predictive algorithms, and experimental validation analyses. Among them, the essential modulatory roles of bone-derived OCN and sclerostin on the brain were highlighted.

Given the current characteristics of various omics technologies, single-cell multi-omics (scMulti-omics) is regarded as the primary methodology for investigating bone-brain comorbidities. By integrating multi-layered molecular information—including genomic, transcriptomic, and chromatin accessibility data—at the single-cell level, this approach surmounts the limitations of conventional single-omics analyses, such as bulk profiling. It provides profound insights into cell-type-specific regulatory networks and disease mechanisms, which are critical for identifying precise biomarkers [298]. Specifically, while bulk genomic analyses yield tissue-level average signals, they fail to resolve regulatory programs specific to distinct cell types or states [299]. Transcriptomic data alone are insufficient to comprehensively capture regulatory mechanisms, including post-translational modifications and genome accessibility. In contrast, scMulti-omics enables the inference of transcription factor-gene interactions across diverse cell types, bridging these knowledge gaps [299]. Meanwhile, proteomics and metabolomics face challenges such as “low specificity in biomarker identification due to overlapping pathologies” and “inconsistent results” [300], highlighting the necessity of multi-omics integration. Although scMulti-omics does not directly tackle protein or metabolite variability, the regulatory networks it unravels can provide a mechanistic framework for these omics—for instance, by guiding targeted analyses of key pathways. Through the integration of scMulti-omics data, molecular interactions across different cellular states can be more comprehensively clarified, thus opening new avenues for mechanistic studies of complex diseases [301].

Current studies have identified several biomarkers of bone-brain inter-regulation. NPY, a key mediator connecting the nervous and skeletal systems, regulates bone formation through osteoblast lineage differentiation while regulating energy metabolism in the central nervous system (especially in the arcuate nucleus of the hypothalamus) [302]. Its expression pattern in peripheral tissues provides a new perspective for the study of the bone-brain axis [303]. The leptin system, on the other hand, exhibits bidirectional regulatory features: Conversely, it enhances synaptic plasticity through hippocampal LepRb receptors [304] and improves spatial memory [305]; on the other hand, it inhibits osteoclastogenesis through STAT3 signaling [306], whose deficiency leads to bone metabolism abnormalities and cognitive decline [307], and is independently predictive of AD risk in human [308]. Notably, supplementation with leptin synchronously elevates bone protective factors such as IGF-1 and estrogen [309], revealing its potential value in coordinating bone-brain function through the hypothalamic-endocrine axis.

Bone-derived hormone studies have further expanded the scope of biomarkers: As an anti-aging factor alleviating cognitive decline [310], OPN is significantly elevated in both postmenopausal osteoporosis and AD pathological processes [311]. Elevated OPN levels in cerebrospinal fluid are associated with early synaptic dysfunction, tau deposition, and neuronal loss in AD, suggesting its potential as a prodromal AD biomarker [311]. Moreover, studies have demonstrated a positive correlation between serum OPN levels and the severity of OP, indicating its utility as an early diagnostic biomarker for postmenopausal osteoporosis [312]. Collectively, these findings imply that OPN may serve as a diagnostic marker for bone-brain comorbidity. Notably, although elevated OPN levels are linked to both diseases, the anatomical sources of measured OPN differ. As a key biomarker of bone metabolism, OCN levels are elevated in osteoporosis [313]. Increased plasma and CSF OCN levels have been observed in preclinical AD, MCI, and AD dementia patients, showing significant associations with cerebral Aβ deposition, pTau, neurodegeneration, and cognitive decline [97]. This implies that elevated OCN levels may facilitate early screening for comorbid AD and osteoporosis. However, prior studies have reported conflicting findings regarding OCN expression in patients with AD, necessitating further clinical and animal studies to elucidate its role in both diseases.

Current evidence suggests that a multidimensional combination of biomarkers integrating bone metabolism indicators (OCN, OPN), NPY and metabolic hormones (leptin), combined with their dynamic network of interactions between central and peripheral, may provide a new strategy for the early identification of bone-brain comorbidities, but the predictive efficacy needs to be validated by longitudinal cohorts, and the specific molecular mechanisms can be explored by combining a multi-omics approach.

The permeability of molecules or drugs across the BBB has long been a focal yet challenging aspect of research on brain-related disorders. Currently, there is a lack of direct studies investigating the penetration of bone-derived factors through the BBB. Based on existing research progress, several well-recognized and highly accurate detection techniques have been employed to predict the permeability of drugs or small molecules across the BBB, including the machine learning-based c-RASAR method [314], biomimetic immobilized artificial membrane chromatography combined with quantitative structure-activity relationship (QSAR) modeling [315], the Parallel Artificial Membrane Permeability Assay-QSAR model [316], and positron emission tomography coupled with computed tomography [317]. Future studies may further explore the penetration efficiency of bone-derived factors across the BBB using these methods, potentially leading to a quantitative assessment of brain function and accelerated repair of brain injuries.

## 8. Conclusions

Bone is not only a motor organ but also an essential endocrine organ. In this paper, we found that bone regulates brain function primarily through its secretions, with a focus on specific cell types. This regulatory function can mitigate brain damage and promote the restoration of brain function. The paper establishes a novel clinical approach by utilizing bone-associated cells as an intervention modality for the treatment of brain diseases, transcending the conventional single-organ study and offering a more comprehensive investigation of the relationship between these two significant organs. Concomitantly, this study will expand our understanding of bone and provide a valuable set of references for the interactions between bone and other organs. The research model can be applied to the study of interactions among other organs, significantly contributing to the comprehension of cell–cell interactions, cross-organ functional regulation, and overall bioregulation.

## Figures and Tables

**Table 1:** Therapeutic and Mechanisms of BMSCs in Brain Diseases.

Diseases	Methods	Effect	Mechanisms
AD	BMSC Transplant, BMSCs-EXO	Amelioration of hippocampal loss and enhancement of memory function in mice [15], Improvement of AD-like behavior in mice [72]	Inhibition of neuronal apoptosis, an increase in hippocampal superoxide dismutase activity, and a significant increase in protein levels of brain-derived neurotrophic factor in the hippocampus [15]. It regulates hippocampal glial cell activation, neuroinflammation, and BDNF [72]. BMSCs-EXO miR-146a can reduce NF-κB levels [80].
Diabetic Cognitive Impairment	BMSC Transplant	Improvement of diabetes-induced cognitive dysfunction [23]	Repair of damaged neurons and astrocyte degeneration [23].
Cerebral ischaemia	BMSCs-NGF	Promoting brain remodeling	Upregulation of neural-like cell differentiation and increased synaptophysin expression [36], downregulates the pro-inflammatory cytokine TNFα [42].
MCAO	BMSCs-VEGF	Improvement in brain function and behavior	Promotes angiogenesis and improves blood flow recovery [46,48].
TBI	BMSCs-EXO	Improves cognitive function and promotes neurological recovery	Reducing glutamate levels [67] promotes neurogenesis, regulates angiogenesis [16], and up-regulation of miR-181b activates the IL10/STAT3 pathway [75] and reduces neuroinflammation [16].
SAH	BMSCs-EXO	Improves neurological function, reduces brain water content, and maintains BBB integrity [69]	Inhibition of NF-κB, activation of AMPK, attenuation of post-SAH inflammation, and neuroprotection [70]. miRNA-129-5p inhibits the anti-inflammatory and anti-apoptotic effects of the HMGB1-TLR4 pathway [69].
PD	BMSCs-EXO	Reversal of rotational behavior and climbing speed in PD mice	Inhibition of the P38MAPK/P65NF-κB signaling cascade and thus the transcription of inflammation-related genes remodels the nigrostriatal inflammatory microenvironment and repairs DA nerve injury [71].

## Data Availability

Not applicable.

## Abbreviations

A cluster of differentiation	CD
Alzheimer’s disease	AD
Blood–brain barrier	BBB
BMSCs-derived exosomes	BMSCs-EXOs
Bone marrow-derived mononuclear cells	BMMs
Bone marrow mesenchymal stem cells	BMSCs
Bone morphogenetic proteins	BMPs
Bone-derived cytokines	BDCs
Brain-derived neurotrophic factor	BDNF
Carboxylated osteocalcin	cOCN
Central nervous system	CNS
Cerebrospinal fluid	CSF
chemokine receptor	Cx3cr1
chronic kidney disease	CKD
C-X-C chemokine receptor type 4	CXCR4
Cyclooxygenase 2	COX2
Early growth response protein 1	Egr1
Exosomes	EXOs
Experimental allergic encephalomyelitis	EAE
Extracellular signal-regulated kinases 1 and 2	ERK1/2
Extracellular vesicles	EVs
Fibroblast growth factor2	FGF2
Fibrosarcoma 1	Raf1
High-mobility group box 1 protein	HMGB1
Hypoxic–ischemic brain damage	HIBD
Inducible nitric oxide synthase	iNOS
Interferon-γ	IFN-γ
Interleukin-1 receptor-associated kinase1	IRAK1
Interleukin-6	IL-6
Intracerebral hemorrhage	ICH
Ischemia/reperfusion	I/R
Ischemic stroke	IS
Klotho	KL
Knockout	KO
Lipocalin-2	LCN2
Malondialdehyde	MDA
Matrix metalloproteinase	MMP
Melanocortin 4 receptor	MC4R
Membrane-bound RANKL	mRANKL
Mesenchymal stem cell	MSC
Multiple sclerosis	MS
Nerve growth factor	NGF
Neuropeptide Y	NPY
Nuclear factor kappa B	NF-κB
Nuclear factor of activated T cells 5	NFAT5
Nuclear factor-κB ligand	RANKL
Osteoblasts	Obs
Osteocalcin	OCN
Osteoclasts	Ocs
Oxygen-glucose deprivation/reperfusion	OGD/R
Parkinson’s disease	PD
PDGF-BB/PDGF receptor	PDGFR β
Permanent middle cerebral artery occlusion	pMCAO
Phosphatase gene	PTEN
Platelet-derived growth factor BB	PDGF-BB
Postsynaptic density protein 95	PSD95
Soluble RANKL	sRANKL
Sphingosine-1-phosphate	S1P
Streptozotocin	STZ
Stromal cell-derived factor 1	SDF-1
Subarachnoid hemorrhage	SAH
Superoxide dismutase	SOD
Tartrate-resistant acid phosphatase positive	TRAP+
The common carotid arteries	2VO
Toll-like receptor-4	TLR4
Transient middle cerebral artery occlusion	TMCAO
Traumatic brain injuries	TBI
Tumor necrosis factor-α	TNF-α
Uncarboxylated osteocalcin	ucOCN
Vascular endothelial growth factor	VEGF
Vitamin D	VD
γ-aminobutyric acid	GABA
